# Aromatic Linkers Unleash the Antiproliferative Potential of 3‐Chloropiperidines Against Pancreatic Cancer Cells

**DOI:** 10.1002/cmdc.202000457

**Published:** 2020-09-15

**Authors:** Tim Helbing, Caterina Carraro, Alexander Francke, Alice Sosic, Michele De Franco, Valentina Gandin, Richard Göttlich, Barbara Gatto

**Affiliations:** ^1^ Institute of Organic Chemistry Justus Liebig University Giessen Heinrich-Buff-Ring 17 35392 Giessen Germany; ^2^ Department of Pharmaceutical and Pharmacological Sciences University of Padova Via Francesco Marzolo 5 35131 Padova Italy

**Keywords:** alkylating agents, aromatic chloropiperidines, DNA damage, pancreatic cancer spheroids, structure-activity relationships

## Abstract

In this study, we describe the synthesis and biological evaluation of a set of *bis*‐3‐chloropiperidines (B−CePs) containing rigid aromatic linker structures. A modification of the synthetic strategy also enabled the synthesis of a pilot *tris*‐3‐chloropiperidine (Tri‐CeP) bearing three reactive *meta*‐chloropiperidine moieties on the aromatic scaffold. A structure–reactivity relationship analysis of B−CePs suggests that the arrangement of the reactive units affects the DNA alkylating activity, while also revealing correlations between the electron density of the aromatic system and the reactivity with biologically relevant nucleophiles, both on isolated DNA and in cancer cells. Interestingly, all aromatic 3‐chloropiperidines exhibited a marked cytotoxicity and tropism for 2D and 3D cultures of pancreatic cancer cells. Therefore, the new aromatic 3‐chloropiperidines appear to be promising contenders for further development of mustard‐based anticancer agents aimed at pancreatic cancers.

## Graphical abstract

**As keen as mustard**: Aromatic 3‐chloropiperidines designed to improve the therapeutic potential of mustard‐based DNA alkylating agents have been synthesized and evaluated. These small molecules exhibited a marked anti‐proliferative effect preferentially against BxPC‐3 pancreatic adenocarcinoma cells in 2D and 3D cultures, thus demonstrating themselves to be promising candidates for the further development of sustainable chemotherapeutics active against pancreatic tumors.

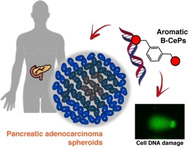

## Introduction

**Figure 1:**
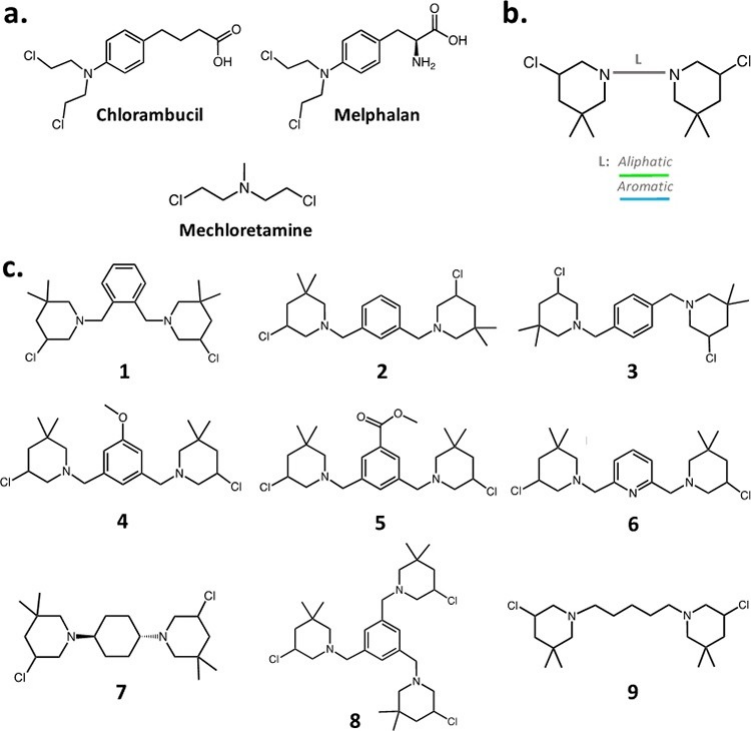
a) Chemical structure of chlorambucil, melphalan and mechloretamine. b) General structure of B−CePs. Different connecting linkers (L) are schematized with different colors. c) Chemical structures of the analyzed 3‐chloropiperidines.

Recent efforts in the search for targeted anticancer agents have led to the discovery of promising candidates that interfere with different carcinogenic pathways. Nevertheless, persistent drawbacks undermine the development of these drugs, such as the rapid emergence of resistance mechanisms and the onerous manufacturing costs, especially for biologics.^[1]^ Consequently, “old” chemotherapeutics still represent a valid choice in first‐line treatments.^[2]^ Among the most diffused alternatives, nitrogen mustards (NMs, such as those depicted in Figure 1a) represent one of the first class of alkylating anticancer agents which act by directly damaging DNA thus preferentially impairing replication and transcription in fast‐dividing tumor cells.^[2]^ Unfortunately, the therapeutic applicability of these drugs is limited by their scarce selectivity, which is frequently associated to tough side effects for patients.[^2^, ^3^] Thus, efforts are required to identify new NM candidates with ameliorated efficacy and safety profiles. Seeking to improve the therapeutic value of these economically sustainable antitumor agents, our studies focused on the development of *bis*‐3‐chloropiperidines (B−CePs) as a new class of revisited mustard‐based alkylating agents (Figure 1b).^[4]^

In preceding works, we thoroughly investigated the mechanism of DNA alkylation by B−CePs. Thanks to their marked electrophilicity, these compounds efficiently react with nucleobases, forming mono‐ and bi‐functional adducts preferentially with the N7 of guanines.^[5]^ These base lesions rearrange into apurinic sites leading to DNA cleavage,^[5]^ with in vitro potencies modulated by the chemistry of the linker connecting the two 3‐chloropiperidine reactive centers.[^4a^, ^4b^] Along with potent derivatives characterized by aliphatic linkers, our previous studies examined the activity toward DNA of one B−CeP bearing a *para*‐xylene linker (compound **3**, Figure 1c).^[4b]^

In vitro, the aromatic rigid linker reduced the DNA cleavage potency relative to the aliphatic analogues, an attribute worth to be further explored in the characterization of this class of compounds.^[4b]^ Similarly, the classical nitrogen mustards bearing aromatic moieties (e. g., chlorambucil and melphalan, Figure 1a) possess a milder electrophilicity, which makes them less toxic drugs compared to aliphatic analogues (e. g., mechlorethamine, Figure 1a).^[6]^

From these premises, the present work aims to further investigate the chemical space and anticancer value of B−CePs by synthesizing and evaluating a series of derivatives bearing different linkers intended at modulating the compounds electrophilicity. The new set is called here for simplicity “*aromatic* B−CePs” as their characteristic feature is the presence of aromatic linkers connecting the two reactive moieties (Figure 1b). The chemical structure of test derivatives is depicted in Figure 1c. In detail, the set includes: i) derivatives with *ortho‐*, *meta‐* and *para‐* xylene linker arrangements (compounds **1**–**3**) to reveal the effects of orientation and distance between reactive centers on the activity of B−CePs; ii) two exploratory derivatives containing electron‐withdrawing functional groups, respectively bearing a methoxy group and a methyl ester function (compounds **4** and **5**) attached in *meta* position to minimize steric effects, in order to probe the possible influence of substituents on the aromatic system; moreover, iii) we incorporated a pyridine linker in compound **6** to study the influence of a heteroatom in the aromatic bridging linker; finally, iv) to probe the value of *tris*‐3‐chloropiperidines (Tri‐CePs), we synthesized compound **8**, bearing three reactive moieties in *meta* on the aromatic scaffold, to explore the correlation between activity and number of reactive centers. For comparative purpose, we included in our analyses the previously synthesized *aliphatic* B−CePs **7**, possessing a rigid aliphatic cyclohexane linker, and **9**, bearing a flexible pentyl linker.^[4b]^

Aside from proposing novel accessible strategies to synthesize the described set of aromatic derivatives, the present work aims at extending their biological evaluation beyond the reactivity on model nucleic acids, investigating the effects of all compounds against different cancer cell types to gain evidence of biological activity and tropism toward different tumors. The remarkable cytotoxicity in the pancreatic tumor‐derived cells prompted us to further explore the valuable biological activity of aromatic B−CePs. We therefore assessed the involvement of transporters in the uptake of B−CePs. Furthermore, we inspected their molecular mechanism of action by checking the presence in cells of genomic DNA lesions upon treatment. Finally, we demonstrated in 3D models of pancreatic cancer how the modulation of reactivity of the aromatic compounds is crucial for unleashing their antitumor potential.

## Results and Discussion

### Synthesis of *bis*‐ and *tris*‐3‐chloropiperidines

**Scheme 1:**
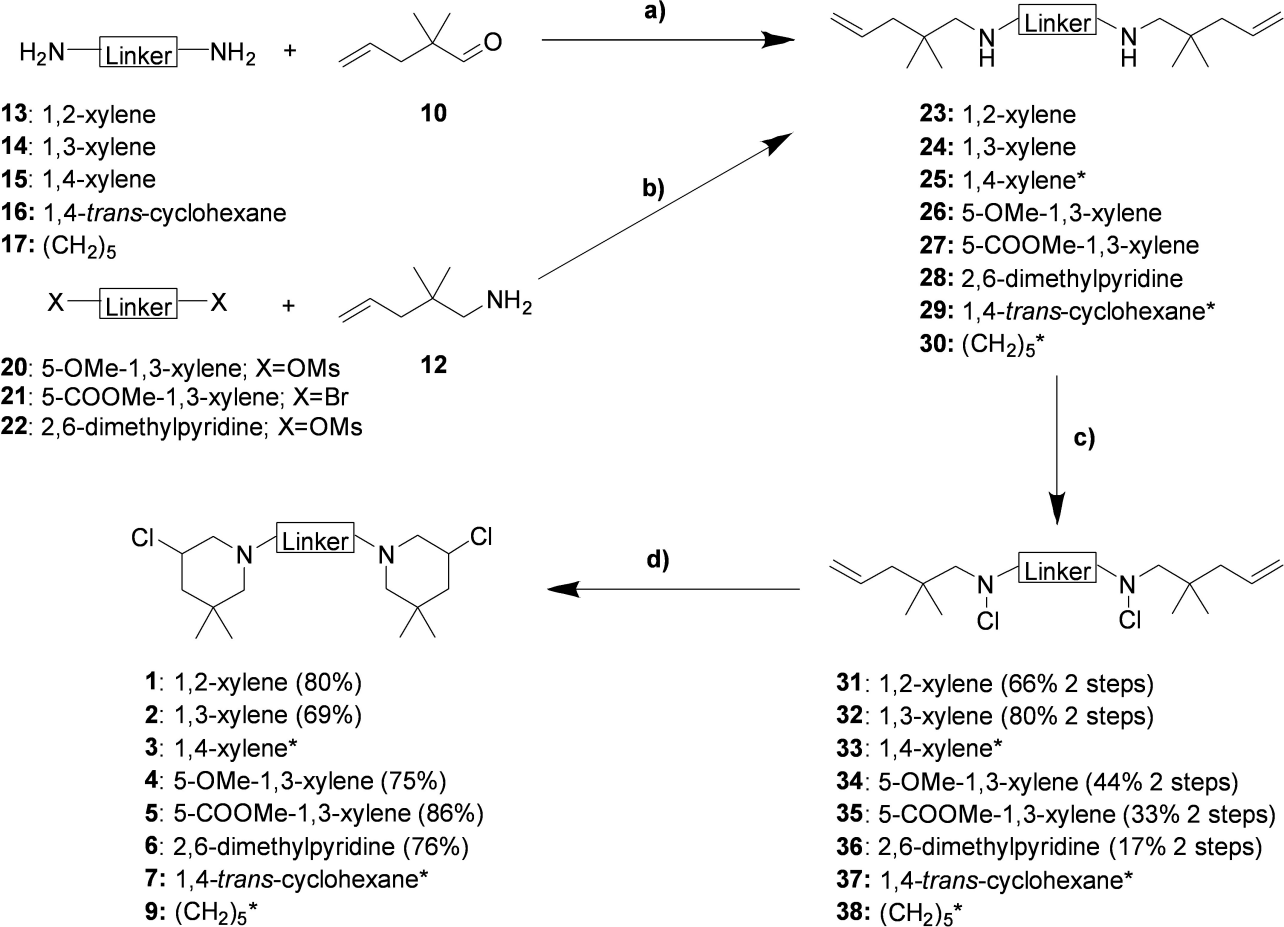
Synthesis of *bis*‐3‐chloropiperidines **1**–**6**. a) NaBH(OAc)_3_, AcOH, dry CH_2_Cl_2_, 0 °C to RT, 16–18 h; b) NaH, dry THF, 0 °C to RT, 20–22 h; c) NCS, dry CH_2_Cl_2_, 0 °C to RT, 2.5–3 h; d) TBAI (cat.), dry CHCl_3_, 60 °C (oil bath temperature), 2–2.5 h (inseparable diastereomeric mixture). *The synthesis of compounds **3**, **7** and **9** as well as their corresponding precursors has been described elsewhere.^[4b]^

The synthetic strategy to obtain the *bis*‐3‐chloropiperidines **1**–**7** and **9** remained similar to our previously reported procedures,^[4]^ but was adapted for the substituted B−CePs **4**–**6** to further extend the range of suitable precursors (Scheme 1). The synthesis of the final products **3**, **7** and **9** has already been described in our previous work.^[4b]^ Starting with the diamines **13**–**17** and the functionalized aromatic building blocks **20**–**22** the desired product was always the corresponding unsaturated diamine **23**–**30**, which was prepared by two different strategies. For the synthesis of the *bis*‐3‐chloropiperidines with varying substitution pattern **1**–**3**, the preparation started with the corresponding aromatic diamines **13**–**15** which were converted to their secondary analogues **23**–**25** by a double reductive amination using 2,2‐dimethylpent‐4‐enal **10** and sodium triacetoxyborohydride.^[7]^ This strategy is well known and was also applied in the previous synthesis of the B−CePs **7** and **9**, starting from their corresponding diamines **16** and **17**.^[4b]^ In contrast, the secondary diamines **26**–**28** were obtained by nucleophilic substitution of a suitable leaving group attached to the substituted aromatic linkers. For this reaction the aldehyde **10** was converted to the corresponding primary amine **12**, which was deprotonated using sodium hydride and then reacted with the brominated or mesylated aromatic precursors **20**–**22**. Afterwards, the secondary diamines **23**–**30** were treated with *N*‐chlorosuccinimide (NCS) to obtain the unsaturated *bis*‐*N*‐chloroamines **31**–**38**. These products were then converted to the desired *bis*‐3‐chlorpiperidines **1**–**7** and **9** by iodine catalyzed cyclization using tetrabutylammoniumiodide.^[8]^

Detailed synthetic procedures for the preparation of the precursors **10**–**13** and **18**–**22** from readily available starting materials can be found in the Supporting Information.

**Scheme 2:**
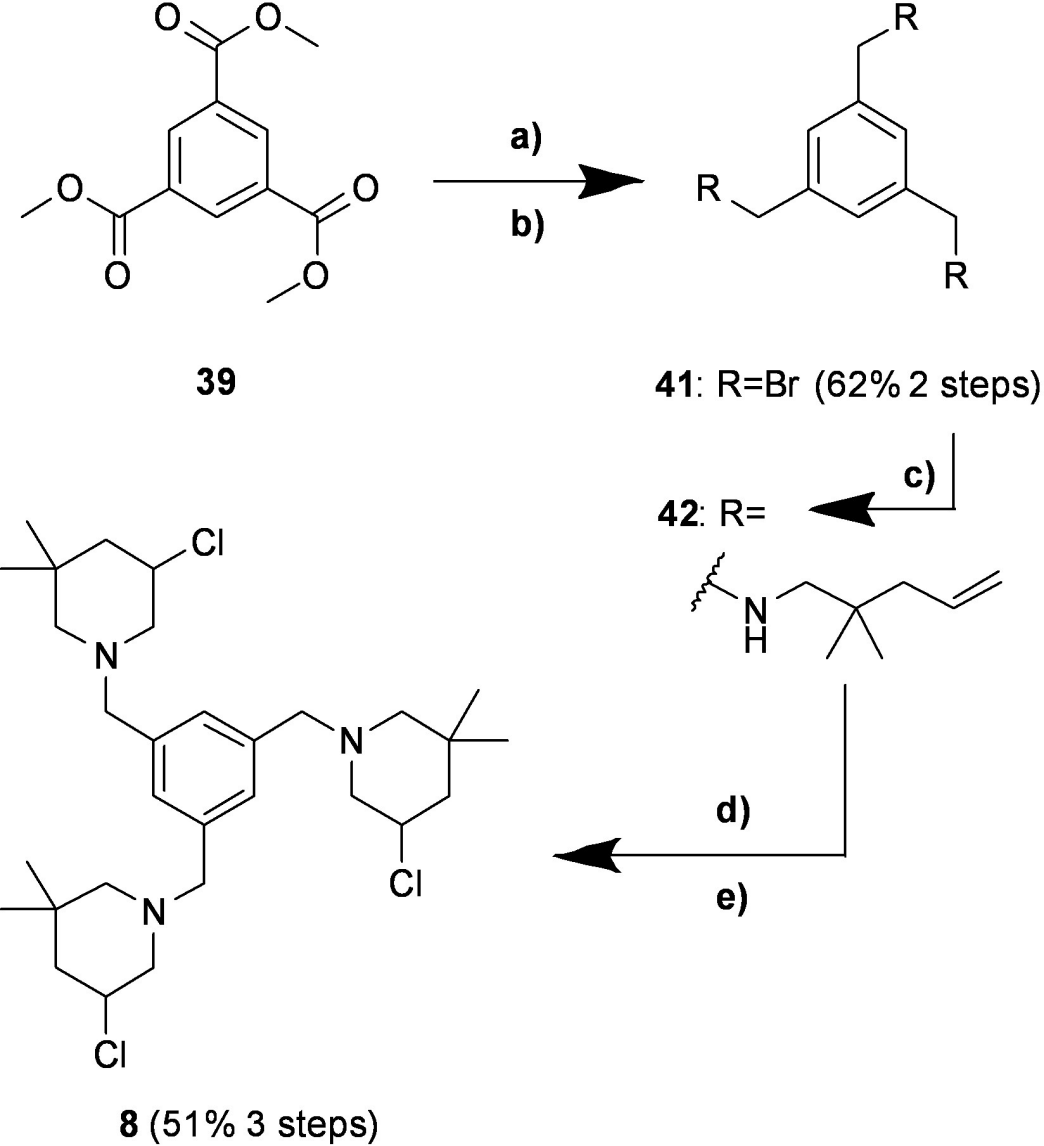
Synthesis of *tris*‐3‐chloropiperidine **8**. a) LAH, dry THF, 0 °C to RT to reflux, 18 h; b) PBr_3_, dry Et_2_O, 0 °C to RT, 24 h; c) 2,2‐dimethylpent‐4‐en‐1‐amine **12**, dry CH_2_Cl_2_, 0 °C to RT, 68 h; d) NCS, dry CH_2_Cl_2_, 0 °C to RT, 2.5 h; e) TBAI (cat.), dry CHCl_3_, reflux, 4 h.

The brominated precursor **41** was synthesized by a two‐step procedure from the readily available trimethyl 1,3,5‐benzenetricarboxylate **39** (see the Supporting Information for details), which was then reacted with an excess of the unsaturated amine **12** to obtain the corresponding secondary triamine **42**. Afterwards, the known synthetic strategy for *bis*‐3‐chloropiperdines, involving the *N*‐chlorination and cyclization, was adopted and applied to the trifunctional compound, providing the desired *tris*‐3‐chloropiperidine **8** in good yield (Scheme 2).

### Aromatic and aliphatic B−CePs exhibit different reactivity

The mechanism of DNA alkylation by B−CePs involves an intramolecular nucleophilic displacement of the chloride in 3‐position, leading to the formation of the reactive aziridinium ion, which is readily attacked by nucleophiles such as water or nucleobases.^[5]^ To support our hypothesis that the reactivity of B−CePs with nucleophiles is influenced by the nature of linker, we determined the kinetics of B−CePs consumption in aqueous solution. We employed electrospray ionization mass spectrometry (ESI‐MS) to monitor the formation of compounds hydrolysis products and intermediates over time. For the aromatic compounds, we analyzed **1** and **2** to investigate the influence of *ortho* and *meta* substitution and compared them with **7** and **9** to explore the effects of aliphatic linkers on the dynamics of B−CePs reactivity. Aqueous solutions of mentioned compounds were incubated at 37 °C and aliquots of the reaction mixtures were analyzed after different incubation times (0, 20, 40, 60, and 180 min and overnight) by ESI‐MS. Graphs showing the relative distribution of detected species (U=unreacted compound, N^+^=aziridinium ion, 2 N^+^=double aziridinium ion, OH=monohydroxylated, N^+^/OH=monohydroxylated/aziridinium ion, 2OH=dihydroxylated) over time are reported in Figure S1 in the Supporting Information. The kinetics of the reaction of aliphatic versus aromatic compounds was very different. Derivatives **7** and **9** were rapidly consumed in water, with the unreacted species coexisting with mono and dihydroxylated species formed at high rates already from 20 minutes of incubation. At 3 h both compounds were almost totally reacted. The reactivity of aromatic compounds was slower compared to the aliphatic analogues, as expected. Besides, we noted the slower kinetics of **1** compared to **2**. The N^+^ species of **2** formed immediately and turned out to be rather stable, with the ratio between unreacted **2** and its N^+^ ion almost preserved during the first hour of incubation, suggesting the presence of a pre‐equilibrium; the dihydroxylated species formed from 60 min up to 3 h. Conversely, compound **1** started reacting with water only after 20 min of incubation leading to the formation of hydroxylated species. Interestingly, the **1** N^+^ intermediate was never detected in time, attesting the different reactivity profile of the *ortho* compared to the *meta* analogue. Based on these findings, we proceeded with the evaluation of reactivity with biological macromolecules.

### *Meta* arrangement increases aromatic B−CePs reactivity with plasmid DNA

**Figure 2:**
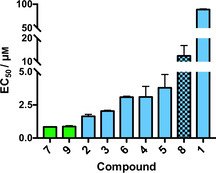
DNA cleavage EC_50_ values of analyzed B−CePs. The supercoiled pBR322 plasmid was incubated with increasing concentrations of test compounds at 37 °C for 3 h in BPE buffer. EC_50_ values were calculated by comparing the intensities of the supercoiled species band to the negative control as a function of compound concentration. Average EC_50_ values and standard deviations result from two independent experiments. Green bars: aliphatic B−CePs; blue bars: aromatic B−CePs; blue checkered bar: Tri‐CeP.

In line with our precedent studies on 3‐chloropiperidines, the new derivatives were initially evaluated for their ability to react with nucleic acids and induce DNA strand breaks.[^4^, ^9^] The electrophoretic cleavage assay allows to visualize the compound‐mediated conversion of the supercoiled plasmid substrate (SC) into its retarded nicked and linearized forms (OC=open circular, L=linearized). In detail, the pBR322 plasmid was incubated with increasing concentrations of test compounds for 3 h at 37 °C and reaction products were run on agarose gel. The bar chart in Figure 2 reports the EC_50_ values of test compounds in ascending order. For the sake of clarity, final EC_50_s of the test derivatives are detailed in Table S1.

With the exception of compounds **1** and **8**, test derivatives exhibited EC_50_ values below 5 μm, thus demonstrating to efficiently cleave DNA in vitro. In line with the previous observations for **3** with a different substrate,^[4b]^ the new results confirm that aromatic linkers (blue bars in Figure 2) decrease the DNA cleavage potency of B−CePs: only the aliphatic compounds **9** and **7** (green bars in Figure 2) possess potencies below 1 μm.

**Figure 3:**
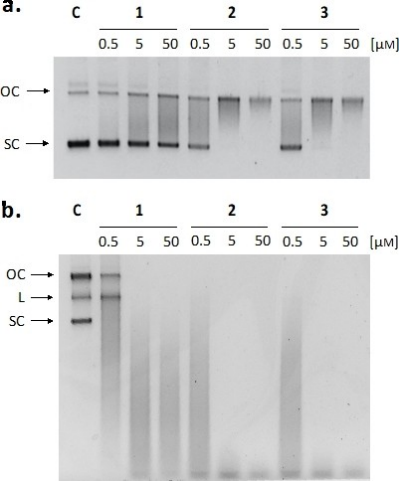
DNA cleavage assay for compounds **1**, **2** and **3**. DNA cleavage assay upon incubation with pBR322 after a) 3 and b) 18 h at 37 °C with increasing compound concentrations (0.5, 5, 50 μm). SC: supercoiled plasmid; L: linearized plasmid; OC: open circular plasmid; C: supercoiled pBR322 plasmid control.

A comparison between the isomers **1**, **2** and **3**, respectively bearing *ortho‐*, *meta‐* and *para‐*xylene linkers, allowed to evaluate the influence of different substitution patterns on the reactivity of aromatic B−CePs. Resulting EC_50_ values demonstrated that, while the *meta* and *para* substitutions conferred a similar potency to compounds **2** and **3**, the activity of the *ortho* positional isomer **1** toward DNA decreased consistently with the reduced reactivity with water. It that conditions, after the first hydroxylation of **1**, intramolecular hydrogen bonding interactions between the newly formed hydroxyl group and the nitrogen atoms of both chloropiperidine rings might reduce the reactivity of the second 3‐chloropiperidine moiety. Such interactions cannot be established when increasing the distance between the two reactive groups as in the case of the *meta* isomer **2**. In addition, the restrained rotability and steric hindrance of **1** may prevent an efficient attack of guanine residues in DNA. Given the limited cleavage of **1** observed at 3 h, the reactivity of **1**, **2** and **3** was indeed investigated at the longer incubation time of 18 h. Resulting gel images are shown in Figure 3a (3 h) and b (18 h). Though much less reactive than its analogues, compound **1** demonstrated to extensively fragment the plasmid at the longer incubation time of 18 h, as clearly attested by the appearance of an intense smear. This suggests that the reaction with nucleic acids still occurs, although it needs time to achieve DNA cleavage compared to the *meta* and *para* isomers. Compounds **4** and **5**, tested to investigate the influence of substitution of the xylene linker, show higher EC_50_ values than **2**, indicating that the insertion of the ether group and the ester substituent reduces the reactivity exhibited by the parent compound. Although there is no direct conjugation to the aromatic system in the case of the substituted B−CePs, the electronic nature of the aromatic system clearly influences the reactivity of the compounds, which decreases in presence of these electron withdrawing groups. As there is no direct conjugation between the piperidine‐nitrogen and the aromatic system, the impact of substituents is limited to inductive effects. Such substituent effects are described by linear free energy relationships derived from the Hammett equation.^[10]^ Though initially developed for substituent effects on the ionization of benzoic acid derivatives, they are also applicable to different organic reactions and have already been applied to aromatic nitrogen mustards.^[11]^ The correlation observed for the substituted B−CePs compared to compound **2** is not perfect, but similar to the reactivity of substituted benzylamines as nucleophiles.^[12]^ This relationship seems to be comparable to our experiments, since the first step in the reaction of B−CePs is always the formation of an aziridiunium ion, provided by a nucleophilic attack of the benzylic piperidine‐nitrogen.

Accordingly, the introduction of a nitrogen heteroatom, reducing the electron density of the aromatic linker in **6**, also decreased the compounds reactivity when compared to analogue **2**. Unintuitively, also the third additional reactive moiety of the Tri‐CeP **8** curtailed its reactivity compared to the bifunctional analogue **2**.

### Aromatic linkers address B−CePs toxicity against pancreatic cancer cells

After analyzing the reactivity of compounds with water and with purified DNA, the biological value of compounds was assessed by the MTT assay on a panel of human cancer cell lines, namely colorectal adenocarcinoma HCT‐15, pancreatic adenocarcinoma BxPC‐3 and ovarian carcinoma 2008 cells. Table 1 reports the IC_50_ values observed after 72 h of treatment with tested B−CePs along with previously determined IC_50_ values of the reference nitrogen mustard chlorambucil (Chl).^[9]^ For its poor solubility and compatibly with the use of maximum 0.5 % DMSO in cell culture, compound **5** was not tested over 25 μm.

**Table 1:** MTT assay IC_50_ values with the associated standard deviations on HCT‐15, BxPC‐3 and 2008 cells of test B−CePs on BxPC‐3 cells after treatment for 72 h.

Compound	MTT IC_50_ values/[μm]		
	HCT‐15	2008	BxPC‐3
**1**	23.8±7.2	16.1±5.6	0.4±0.1
**2**	3.3±1.1	2.8±1.1	0.3±0.2
**3**	3.0±0.9	2.3±0.3	0.4±0.1
**4**	9.5±2.7	4.0±1.9	0.5±0.2
**5**	>25	>25	0.4±0.1
**6**	14.2±2.2	10.7±2.7	0.4±0.1
**7**	3.2±2.5	2.6±0.8	5.5±1.1
**8**	9.3±2.9	14.3±5.5	0.3±0.2
**9**	6.8±2.1	11.3±2.2	9.4±1.6
**Chl^[a]^**	49.7±3.3	12.5±2.1	75.3±5.1

[a] Reference IC_50_ values reported in our previous work^.[9]^ Chl: Chlorambucil. IC_50_ values were calculated by a four‐parameter logistic model.

Of note, all derivatives demonstrated a valuable cytotoxicity against the three human tumor cell lines, in most cases improved compared to the reference drug chlorambucil.^[9]^ Interestingly, results suggest a separate analysis for HCT‐15 and 2008 compared to BxPC‐3 tumor cells: in fact, B−CePs behaved similarly against colorectal and ovarian cancer cells, exhibiting IC_50_ values between 1 and 20 μm, whereas the new set was one or two orders of magnitude more potent against the pancreatic cancer cells. Considering the former two cell lines, aromatic derivatives exhibit a positive correlation between cytotoxicity and reactivity. The poorly reactive isomer **1** bearing the *ortho*‐xylene linker turned out to be less cytotoxic than its *meta*‐ and *para*‐analogues **2** and **3**. The ether and ester substituents led to a decrease both in the reactivity and cytotoxicity of **4** and **5** compared to compound **2**, following the ranking **2**>**4**>**5**. Moreover, compound **2** resulted more cytotoxic and more reactive than its pyridine analogue **6**. Finally, the bifunctional agent **2** was also more cytotoxic than the trifunctional derivative **8**, always in line with cleavage results. However, when considering the aliphatic derivatives **7** and **9**, it is evident that the overall reactivity and cytotoxicity rankings do not match: unsurprisingly, this suggests that factors other than reactivity finally contribute to determine the cellular activity of B−CePs. When considering the aromatic series, we can conclude that for the colorectal and ovarian cancer models our results corroborate the mechanism of action of this class of alkylators and confirm the feasibility of modulating the activity of B−CePs by acting on the chemistry of the linker.

Results concerning the pancreatic cell line are quite divergent: very interestingly, all and only the *aromatic* derivatives showed nanomolar IC_50_ values against pancreatic tumor cells, showing to be on average 200 times more toxic for BxPC‐3 than chlorambucil, a difference not so evident considering HCT‐15 and 2008 cells.

**Figure 4:**
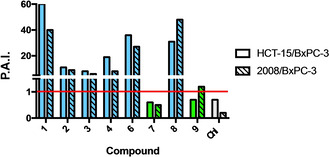
Preferential activity indexes of 3‐chloropiperidines. P.A.I are expressed as the ratio between the compound MTT IC_50_ against HCT‐15 and BxPC‐3 cells (solid bars), as well as 2008 and BxPC‐3 cells (dashed bars). Chl: Chlorambucil. Green bars: aliphatic B−CePs; blue bars: aromatic B−CePs; gray bars: Chl.

To highlight the unexpected tropism toward the pancreatic cancer cell line, the preferential activity index (P.A.I.) expressed as ratio between the IC_50_ of each compound against 2008 or HCT‐15 relative to BxPC‐3 cells is reported in Figure 4.

Notably, we observe a marked selectivity for the pancreatic cell line (Figure 4), with the least DNA reactive compounds **1** and **8** among the most selective for BxPC‐3 cells. On the other hand, the aliphatic compounds **7** and **9** (green bars in Figure 4) showed no different behavior throughout the screened panel, resulting in no selectivity and much higher IC_50_ values for pancreatic cancer cells.

Given the encouraging cytotoxicity observed against the pancreatic cancer cell line, we proceeded to better characterize the biological profile of the tested compounds.

### B−CePs exploit transporter‐mediated uptake

**Figure 5:**
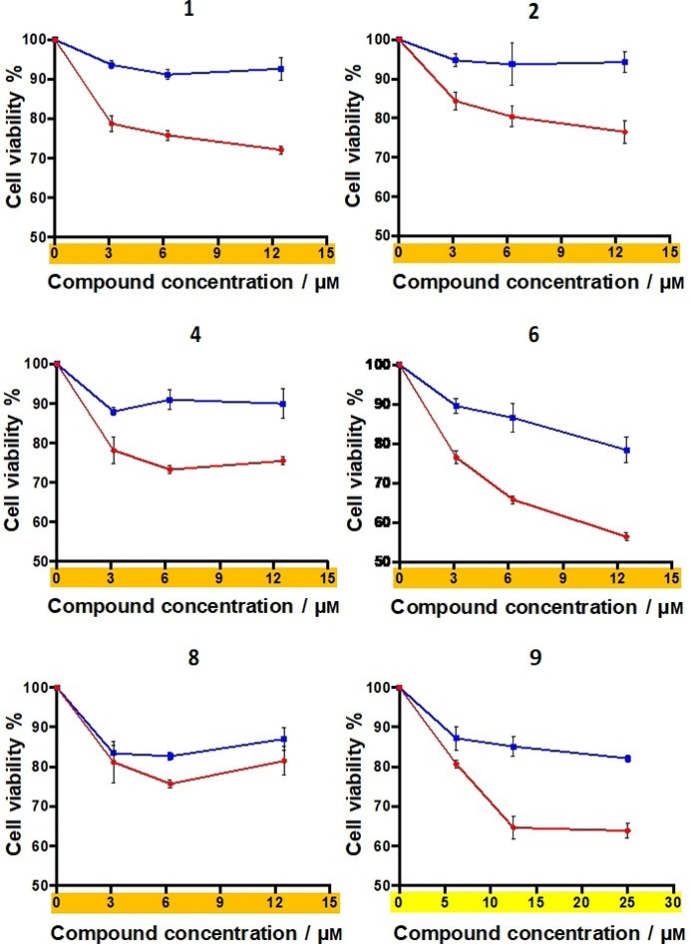
BxPC‐3 cell viability upon exposure to increasing concentrations of selected compounds (3 to 12 μm of rigid derivatives **1**, **2**, **4**, **6**, **8** and 10 to 25 μm of **9**) for 5 h at 37 °C (•) or 4 °C (▪), followed by a gentle rinse of wells with PBS and addition of fresh RPMI medium. The MTT assay was performed at 72 h.

A modified MTT assay was performed on selected derivatives to investigate their mode of entry in BxPC‐3 cells to disclose possible mechanisms of transporter‐mediated uptake.^[13]^ We analyzed the aromatic B−CePs **1**, **2**, **4**, **6** together with compound **9** as representative of the aliphatic subset. The BxPC‐3 selective trifunctional compound **8** was also examined. Given the almost equivalent reactivity and cytotoxicity profile between **2** and **3**, the latter compound was not included in this analysis. Besides, being a non‐optimal candidate for its poor solubility, compound **5** was not further investigated. Cells were seeded in two microplates and incubated for 5 h in parallel at 37 or 4 °C. Given the short incubation time, the adopted range of concentrations was increased compared to the IC_50_ values obtained at 72 h. At the end of the incubation time the wells were rinsed with PBS, fresh medium was added and both microplates were incubated at 37 °C in the absence of compound. Finally, cell viability was assessed at 72 h and resulting curves are shown in Figure 5.

The differential viability resulting from the incubation of *twin* microplates at 37 or 4 °C allows to estimate the relative contribution of transporters to the net intracellular accumulation of the compounds. The formazan absorbance of untreated controls was almost identical between plates incubated at 37 and 4 °C, suggesting that the sole initial temperature gap was not responsible for differences in viability, measured as metabolic state. At 37 °C (red line), both passive diffusion and transporter‐mediated uptake could contribute to the net accumulation of cytotoxic agents in cells. On the other hand, the cytotoxicity observed at 4 °C (blue line) is almost exclusively a result of the passive diffusion of compounds across the cell membrane, since transporters are inhibited at low temperatures. A substantial drop in cell viability was observed upon treatment with all B−CePs derivatives at 37 °C compared to 4 °C (Figure 5), suggesting a key role for membrane transporters in determining intracellular accumulation of tested B−CePs. Nevertheless, the residual cytotoxicity observed at 4 °C demonstrates that compounds are also capable of passively diffusing through the cell membrane.

Interestingly, a comparable uptake profile was highlighted between **2** and **9**, respectively aromatic and aliphatic analogues. This evidence confutes the hypothesis that the tropism toward BxPC‐3 cells observed for aromatic but not aliphatic derivatives could depend on an enhanced cell penetration of the former. Results suggest that the pyridine linker enhances the intracellular accumulation of **6** by fostering its passive diffusion. Interestingly, the third reactive moiety of **8** apparently enhances its passive diffusion while clearly decreasing its transporter‐mediated uptake compared to other bifunctional analogues.

### Aromatic B−CePs directly damage cellular DNA

**Figure 6:**
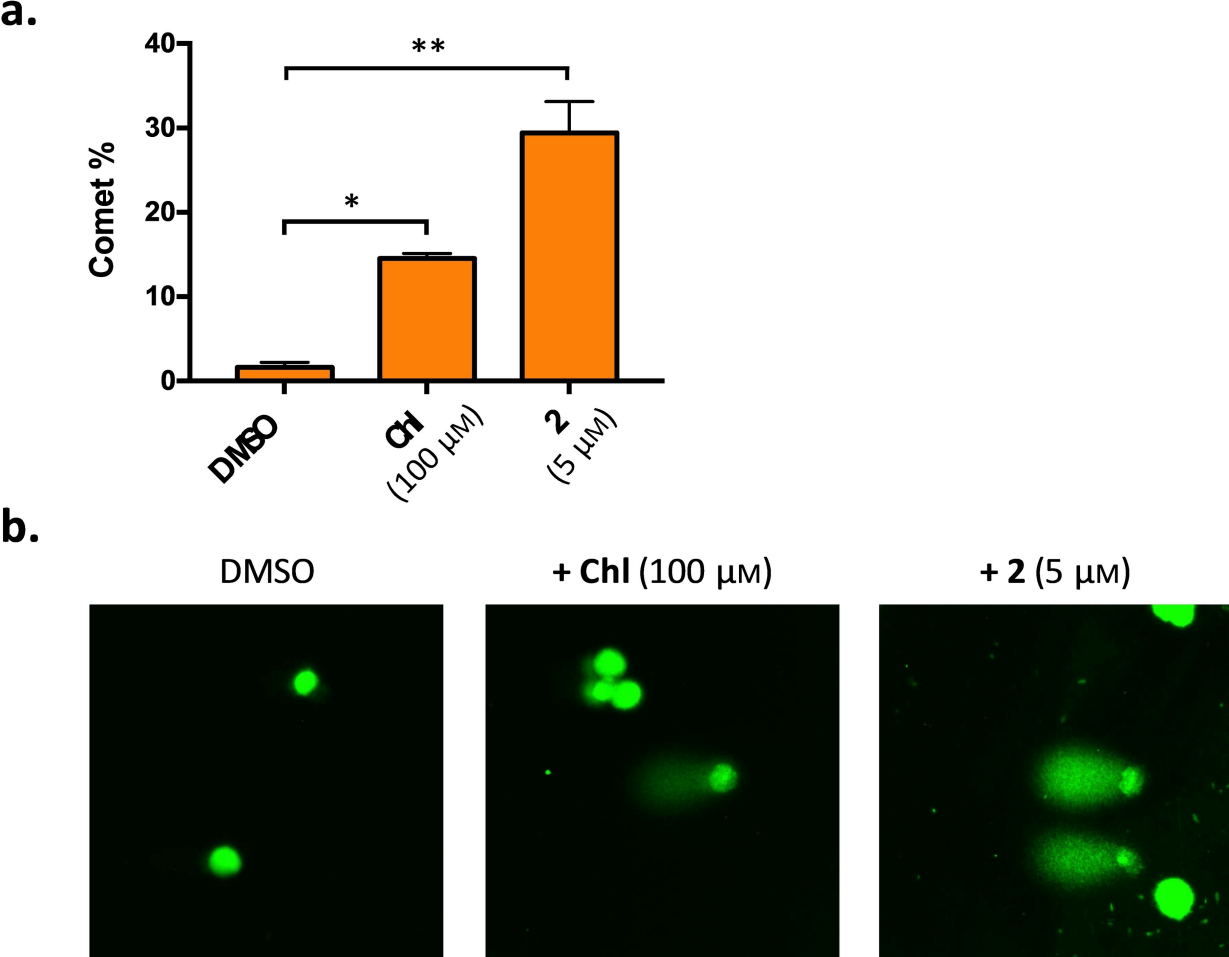
An alkaline SCGE assay was performed on BxPC‐3 cells upon incubation with DMSO 0.5 %, chlorambucil (Chl) and compound **2** for 6 h at the indicated concentrations. a) The relative percentage of comets (number of cells forming a comet/total number of cells) detected in two randomly captured fields from two independent experiments per condition. b) Representative images (40x) of treated samples (including negative and positive controls) with comets are also reported. Paired t‐test: * *p*<0.05, ** *p*<0.001.

In support of the activity observed on isolated DNA and as proof of principle of their mechanism of action, the alkaline single‐cell gel electrophoresis (SCGE) assay was performed to evaluate potential genomic DNA lesions induced by B−CePs in cells.^[14]^ The highly cytotoxic derivative **2** was chosen as representative of the aromatic B−CePs set. BxPC‐3 cells were exposed for 6 h to 5 μm of compound **2**: this short incubation time allows observing only direct compound‐mediated DNA damage (DNA strand breaks, alkaline‐labile sites), while excluding apoptotic fragmentation. Lesioned genomic DNA subjected to alkaline treatment and electrophoresis leads to the appearance of a characteristic comet tail upon staining with SYBR Green I. The same experiment was performed in presence of 0.5 % DMSO and of the reference drug chlorambucil (Chl) at the concentration of 100 μm, representing the negative and positive controls respectively. Figure 6 shows the results of the SCGE assay: Figure 6a reports the relative percentage of comets, that is, the number of cells forming a comet relative to the total number of cells detected in two randomly captured fields from two independent experiments per each condition, while Figure 6b reports representative images of the comets in each condition.

Compound **2** harshly damages cellular DNA: in fact, upon 6 h of incubation with 5 μm of compound **2**, at least one third of treated cells gave rise to a fragmented comet. This phenomenon was much reduced in case of the positive control chlorambucil: even at the much higher concentration of 100 μm, only 15 % of cells nuclei got damaged by the drug, and the damage was less severe, as attested by the representative images reported in Figure 6b. Overall, these cellular evidences endorse the mechanism of action of this class of DNA alkylators and justify the significant difference in cytotoxicity observed between our new set of derivatives and chlorambucil.

### 3‐Chloropiperidines exhibit remarkable cytotoxicity against pancreatic cancer spheroids

In light of the promising cytotoxicity observed against BxPC‐3 2D monolayer cultures, the activity of the aromatic B−CePs was further evaluated on 3D BxPC‐3 spheroids, a more sophisticated model for the in vitro screening of therapeutics that better approximates the milieu and hypoxic core of solid tumors.^[15]^ The acidic phosphatase assay (APH) was performed to assess viability of BxPC‐3 spheroids grown in 96‐well round‐bottom microplates for two days and then incubated with selected compounds for 72 h.^[16]^ The APH IC_50_ values for test derivatives are reported in Table 2.

**Table 2:** BxPC‐3 3D cultures cytotoxicity.

Compound	APH IC_50_ values/[μm]	Compound	APH IC_50_ values/[μm]
	BxPC‐3		BxPC‐3
**1**	40.1±7.6	**4**	46.2±0.6
**2**	122.4±5.7	**6**	112.7±2.4
**3**	133.1±4.4	**8**	68.2±4.6
**Chl**	inactive

APH assay IC_50_ values with the associated standard deviations of selected compounds on BxPC‐3 cancer cell 3D cultures after treatment for 72 h. IC_50_ values were calculated by a four‐parameter logistic model. Chl: Chlorambucil, totally inactive at the maximum tested concentration (200 μm).

Aromatic B−CePs confirmed their cytotoxicity also against BxPC‐3 spheroids. Passing from 2D to 3D cultures, we can appreciate more pronounced inter‐compound differences: in spheroids, compound **1** bearing the *ortho*‐xylene linker was the most cytotoxic, followed by **4**, bearing the *meta*‐methoxy xylene linker, and by the new Tri‐CeP agent **8**. Compounds **2**, **3** and **6** exhibited IC_50_ values over 100 μm, a relevant result considering that chlorambucil was completely inactive up to the maximum tested concentration of 200 μm. To understand this result, we should reason about the requisites necessary for compounds to be effective against spheroids. Being three‐dimensional, these systems are constituted by stratified cell layers and we can speculate that B−CePs must efficiently penetrate them while keeping their reactivity intact for a sufficient time. In this sense, a lower or delayed reactivity and a good uptake profile turn out to be crucial for the biological activity of these new set of alkylators in 3D models. Compound **1** was the least reactive with nucleophiles but could demonstrate effective time‐dependent DNA damaging activity (Figure 3b). Compound **8** showed also a restrained reactivity at the DNA cleavage assay but it demonstrated to almost exclusively enter cells by passive diffusion, a slow and less efficient process. Noteworthy, **1** and **8** were highlighted to be among the most selective against the pancreatic cancer cell line (Figure 4), thus showing to be promising leads for upgraded studies as anticancer agents for pancreatic tumors. Although more reactive than **1** and **8**, the methyl‐ether compound **4** showed a valuable activity against spheroids: this result might depend on the ability of the ether function to hamper potential self‐stacking interactions between aromatic molecules, thus increasing the efficiency of spheroid penetration.

## Conclusions

This study highlighted the anticancer potential of a new set of 3‐chloropiperidines designed to improve the efficacy of mustard‐based chemotherapeutics obtained through a convenient synthetic strategy. Different linker substituents and substitution patterns demonstrated to modulate the reactivity of B−CePs toward isolated DNA. Most interestingly, *meta‐* and *para*‐xylene isomers demonstrated to provide a similar and efficient potency of alkylation on isolated DNA, while the *ortho* analogue and B−CePs incorporating electron withdrawing substituents on the aromatic linker exhibited a delayed reactivity. Noteworthy, we demonstrated for the first time the potent antiproliferative effect of B−CePs on a panel of tumor cell lines. In the case of aromatic B−CePs, a clear correlation between reactivity and cytotoxicity was observed against ovarian 2008 and colorectal HCT‐15 cancer cells. Unexpectedly, aromatic but not aliphatic linkers confer to B−CePs nanomolar toxicities against pancreatic BxPC‐3 cells, resulting into valuable indexes of preferential activity for this cancer cell line. As proof of principle of their mechanism of action, the representative aromatic derivative **2** demonstrated to damage cellular DNA to a higher extent compared to chlorambucil even at a much lower concentration. The tropism toward pancreatic cancer cells is exceptionally relevant considering the aggressiveness of this tumor type and recalls what observed with monofunctional 3‐chloropiperidines (M–CePs) against the same cell line.^[9]^

In this study we also investigated the new trifunctional 3‐chloropiperidine **8**, which showed a valuable activity against the advanced model of pancreatic 3D spheroid cultures, most likely by virtue of its lower reactivity, a feature shared with the low reactive B−CeP **1**. This attests that restrained reactivity and good uptake are determinants for the effectiveness of 3‐chloropiperidines in this model, a precious knowledge for the potential development of such compounds against solid tumors.

To conclude, the empowered anticancer features of aromatic B−CePs compared to aliphatic analogues provide a versatile starting point for the advanced synthesis of such compounds. This exclusivity seems not to rely on differences in the intracellular accumulation of aromatic compared to aliphatic compounds, since both showed a very similar uptake profile. Nevertheless, studies are ongoing to reveal which distinctive mechanisms prior or following DNA damage are responsible for the unexpected activity of aromatic B−CePs against BxPC‐3 pancreatic cancer cells. Preferential targeting by nitrogen mustards has recently been reported for chlorambucil against BRCA 1/2‐deficient tumors, thus demonstrating that even agents commonly known as *aspecific* could exhibit cancer type tropisms.^[17]^ In this sense, we plan to employ an omic approach to elucidate the molecular determinants of sensitivity for aromatic 3‐chloropiperidines against BxPC‐3 cells. Perturbations of the transcriptional profile and chromatin accessibility state will be evaluated upon cell stimulation with selected derivatives. This signature‐driven strategy may open new perspectives in the refinement and exploration of the mechanism of action of these new chemical entities, facilitating the mindful repositioning of B−CePs as potential therapeutics.

## Experimental Section

### Materials and Methods

**MS studies**. The formation of B−CePs reactive species upon incubation at 37 °C in water was followed in time by ESI‐MS as reported previously.^[4a]^ A 80 μm solution of test compounds was prepared in MilliQ water from 1 mm DMSO stocks and incubated at 37 °C for 3 h. Small samples were taken after 0, 20, 40, 60 and 180 min, diluted 1 : 10 with methanol, and analyzed by ESI‐MS. Measurements were performed in positive ion mode using a Xevo G2‐XS Qtof instrument (Waters).

**Cleavage assay**. The ability of compounds to cleave the supercoiled plasmid pBR322 (Inspiralis Ltd) was investigated at the electrophoretic cleavage assay. The assay was performed following previously reported materials and protocols.[^4a^, ^4b^] B−CePs dilutions were freshly prepared from a 10 mm DMSO stock in Milli‐Q water, resulting in a 0.5 % DMSO concentration in the final reaction volume. Diversely, a 5 mm DMSO stock was prepared in the case of the poorly soluble compound **5**, resulting in a final 1 % DMSO concentration in the assay volume. EC_50_ values were calculated considering the intensity of the supercoiled plasmid band at increasing compound concentrations expressed as percentage compared to the untreated control. Experiments were performed in duplicate to calculate average values and standard deviations.

**Cell cultures**. Colon (HCT‐15) and pancreatic (BxPC‐3) carcinoma cell lines were purchased from American Type Culture Collection (ATCC). The human ovarian 2008 cancer cell line was kindly provided by G. Marverti (Department of Biomedical Science, University of Modena and Reggio Emilia, Modena, Italy). Cell lines were maintained in logarithmic phase at 37 °C in a 5 % carbon dioxide atmosphere using RPMI‐1640 medium (Euroclone) containing 10 % fetal calf serum (FCS, Euroclone), antibiotics (50 units/mL penicillin and 50 μg/mL streptomycin), and 2 mm L‐glutamine.

**MTT assay**. The 72 h MTT assay was performed following previously reported materials and protocols.[^9^, ^18^] Test compounds were dissolved in DMSO and added at defined concentrations to the cell growth medium to a final solvent concentration of 0.5 %, which had no detectable effect on cell killing. A modified MTT assay was employed to investigate the uptake of test compounds. BxPC‐3 cells were incubated either in the absence or presence of the compound for 5 h at 37 or 4 °C in parallel microplates. At the end of the incubation, fresh RPMI was added to wells replacing the medium containing the compound. Microplates were then incubated both at 37 °C up to 72 h and the MTT assay was performed. The method was validated in this modified version using cisplatin as positive internal control (Figure S2). This drug is known to exploit transporters to enter cells.[^13^, ^19^]

**Alkaline single‐cell gel electrophoresis**. The alkaline SCGE assay allowed to investigate the possible direct damage of genomic DNA induced by B−CePs on BxPC‐3 cells. Experiments were performed following a previously reported protocol with only minor modifications.^[20]^ 3×10^5^ cells were seeded in 25 cm^2^ flasks and incubated after 24 h with the defined concentration of compound or 0.5 % DMSO for 6 h. Subsequently, cells were washed in PBS, harvested, centrifuged and resuspended at 1×10^5^ in 1 % low melting point agarose (LMPA, Trevigen). Pretreated comet slides (Trevigen) were spotted with 50 μL of cells‐LMPA mixture and allowed to set at 4 °C for 30 min. Slides were immersed in lysis buffer (Trevigen) for 45 min, then incubated for 20 min in an alkaline electrophoresis solution (1 mm EDTA, 300 mm NaOH) followed by alkaline electrophoresis (1 V/cm) at 4 °C for 30 min. Slides were then washed twice in deionized H_2_O, fixed in 70 % ethanol for 5 min and air‐dried. DNA was stained with SYBR Green I for 10 min at 4 °C. Slides were examined at 5x and 40x magnification in a Zeiss LSM 800 confocal microscope using the Zeiss ZEN 2.3 software system. The relative % of comets (no. cells forming a comet/ total no. of cells) detected in two randomly captured fields (including at least 250 cells/field) from two independent experiments per condition was analyzed.

**Acid phosphatase (APH) assay**. The APH assay was employed to assess cell viability of spheroids treated with test compounds following previously reported protocols.^[16]^ BxPC‐3 cells were seeded (1.5×10^3^ cells/well) in phenol red‐free RPMI medium (Euroclone) containing antibiotics and l‐glutamine (as previously specified) as well as 10 % FCS and 20 % methyl cellulose in round‐bottom non‐tissue culture treated 96 well‐plates (Greiner Bio‐one). After 72 h from seeding, spheroids were treated at the defined concentration of compound freshly dissolved in DMSO to a final solvent concentration of 0.5 %, which had no detectable effect on cell killing. Upon 72 h of incubation, spheroids were incubated with 100 μL of the assay buffer for 3 h at 37 °C (0.1 m sodium acetate, 0.1 % Triton‐X‐100, supplemented with ImmunoPure *p*‐nitrophenyl phosphate by Sigma). Subsequently, 10 μL of 1 m NaOH were added before measuring the absorbance of each well at 405 nm (BioRad 680 microplate reader). Compounds IC_50_ values were extrapolated applying a four‐parameter logistic (4‐PL) model and average values of two experimental replicates were reported.

### Chemistry

All solvents were purified by distillation prior to use and in case of anhydrous solvents dried and stored under nitrogen atmosphere. Commercially available reagents were used as supplied if not stated different. Synthesis using anhydrous solvents were carried out under Schlenk conditions. For purification by flash column chromatography silica gel 60 (Merck) was used. ^1^H and ^13^C NMR spectra were recorded at Bruker Avance II 200 spectrometer (^1^H at 200 MHz; ^13^C at 50 MHz) and Bruker Avance II 400 spectrometer (^1^H at 400 MHz; ^13^C at 100 MHz) in deuterated solvents. Chemical shifts were determined by reference to the residual solvent signals. High‐resolution ESI mass spectra were recorded in methanol using a ESImicroTOF spectrometer (Bruker Daltonics) in positive ion mode. All elemental analysis (CHN) were performed on a Thermo FlashEA‐1112 series instrument. NMR spectra of all final products, as well as the synthetic procedures of the precursors **10**–**13**, **18**–**22** and **40**–**43** are included in the Supporting Information. The synthesis of compounds **3**, **7** and **9** as well as their corresponding precursors has been described elsewhere.^[4b]^

### Synthetic procedures

#### General procedure A: Synthesis of diamines (23–24)

Under a nitrogen atmosphere 2,2‐dimethylpent‐4‐enal (**10**; 2.1–2.4 equiv) as well as the corresponding diamine were dissolved in anhydrous dichloromethane (10 mL/mmol of diamine) and sodium triacetoxyborohydride (2.6–3 equiv) was added portion wise at 0 °C, followed by acetic acid (2.2–2.4 equiv). The mixture was stirred at room temperature for 16–18 h and was then quenched by the addition of 20 % NaOH solution. The phases were separated and the aqueous layer was extracted three times with dichloromethane. The combined organic extracts were washed with brine, followed by distilled water and dried over MgSO_4_. The solvent was removed under reduced pressure and the crude product was obtained, which was used in the next step without further purification.

#### 1,2‐Bis‐[(2,2‐dimethylpent‐4‐enyl)aminomethyl]benzene (23)

Was prepared according to the general procedure **A** from 1,2‐*bis*(aminomethyl)benzene (**13**; 0.71 g, 5.23 mmol) and 2,2‐dimethylpent‐4‐enal (**10**; 1.41 g, 12.55 mmol) yielding the title compound as a pale red oil (1.50 g, 4.58 mmol). ^1^H NMR (400 MHz, CDCl_3_): *δ*=7.33–7.29 (m, 2H), 7.26–7.22 (m, 2H), 5.84–5.74 (m, 2H), 5.03–4.96 (m, 4H), 3.83 (s, 4H), 2.41 (s, 4H), 2.01 (d, *J*=7.5 Hz, 4H), 0.89 (s, 12H) ppm; ^13^C NMR (101 MHz, CDCl_3_): *δ*=139.21, 135.65, 129.78, 127.19, 116.96, 60.33, 52.93, 44.86, 34.54, 25.69 ppm; HRMS (ESI): *m/z* calcd for C_22_H_37_N_2_^+^: 329.2951; found: 329.2950 [*M*+H]^+^.

#### 1,3‐Bis‐[(2,2‐dimethylpent‐4‐enyl)aminomethyl]benzene (24)

Was prepared according to the general procedure **A** from 1,3‐*bis*(aminomethyl)benzene (**14**; 1.03 g, 7.56 mmol) and 2,2‐dimethylpent‐4‐enal (**10**; 1.75 g, 15.60 mmol) yielding the title compound as a colorless oil (2.18 g, 6.64 mmol). ^1^H NMR (200 MHz, CDCl_3_): *δ*=7.42–7.28 (m, 4H), 5.90–5.65 (m, 2H), 5.11–4.93 (m, 4H), 4.13 (s, 4H), 2.93 (s, 4H), 2.08 (d, *J*=7.2 Hz, 4H), 0.95 (s, 12H) ppm; ^13^C NMR (50 MHz, CDCl_3_): *δ*=137.79, 135.33, 130.18, 128.80, 128.39, 117.43, 73.42, 70.49, 45.03, 35.75, 25.95 ppm; HRMS (ESI): *m/z* calcd for C_22_H_37_N_2_^+^: 329.2951; found: 329.2958 [*M*+H]^+^.

#### General procedure B: Synthesis of diamines (26–28)

Under a nitrogen atmosphere sodium hydride (2.8 equiv) was suspended in anhydrous tetrahydrofuran (10 mL/100 mg of sodium hydride) and 2,2‐dimethylpent‐4‐en‐1‐amine (**12**; 2.4 equiv) was added dropwise at 0 °C. The mixture was stirred at 0 °C for 30 min and the corresponding dibromide or *bis*‐mesylate, dissolved in anhydrous tetrahydrofuran (2 mL/mmol of dibromide/*bis‐*mesylate), was added dropwise at 0 °C. Afterwards the mixture was stirred for 1 h at 0 °C and for an additional 20 h at room temperature. The reaction was then quenched by the addition of 10 % NaOH solution. The phases were separated and the aqueous layer was extracted three times with ethyl acetate. The combined organic extracts were washed with distilled water, followed by brine and dried over MgSO_4_. The solvent was removed under reduced pressure and the crude product was obtained, which was used in the next step without further purification.

#### 5‐Methoxy‐1,3‐*bis*‐[(2,2‐dimethylpent‐4‐enyl)aminomethyl]‐benzene (26)

Was prepared according to the general procedure **B** from 5‐methoxy‐1,3‐bis‐[(methylsulfonyloxy)methyl]benzene (**20**; 2.50 g, 7.71 mmol) and 2,2‐dimethylpent‐4‐en‐1‐amine (**12**; 2.09 g, 18.46 mmol) yielding the title compound as a yellow oil (2.71 g, 7.55 mmol). ^1^H NMR (400 MHz, CDCl_3_): *δ*=6.92 (s, 1H), 6.84 (s, 2H), 5.81–5.73 (m, 2H), 5.03–4.98 (m, 4H), 3.82–3.81 (m, 7H), 2.40 (s, 4H), 2.04–2.02 (m, 4H), 0.91 (s, 12H) ppm; ^13^C NMR (101 MHz, CDCl_3_): *δ*=160.04, 135.39, 120.61, 117.19, 112.60, 59.35, 55.42, 54.41, 44.72, 34.37, 25.57 ppm; HRMS (ESI): *m/z* calcd for C_23_H_39_N_2_O^+^: 359.3057; found: 359.3053 [*M*+H]^+^.

#### Methyl 3,5‐*bis*‐[(2,2‐dimethylpent‐4‐enyl)aminomethyl]‐benzoate (27)

Was prepared according to the general procedure **B** from 3,5‐*Bis*‐(bromomethyl)benzoic acid methyl ester (**21**; 1.17 g, 3.63 mmol) and 2,2‐dimethylpent‐4‐en‐1‐amine (**12**; 0.99 g, 8.72 mmol) yielding the title compound as a yellow oil (1.38 g, 3.56 mmol). ^1^H NMR (400 MHz, CDCl_3_): *δ*=7.89–7.85 (m, 2H), 7.58–7.53 (m, 1H), 5.83–5.73 (m, 2H), 5.03–4.97 (m, 4H), 3.91 (s, 3H), 3.83 (s, 4H), 2.35 (s, 4H), 2.02 (d, *J*=7.5 Hz, 4H), 0.89 (s, 12H) ppm; ^13^C NMR (101 MHz, CDCl_3_): *δ*=167.45, 141.33, 135.60, 132.53, 130.33, 127.93, 117.01, 59.67, 54.34, 52.22, 44.76, 34.50, 25.67 ppm; HRMS (ESI): *m/z* calcd for C_24_H_39_N_2_O_2_^+^: 387.3006; found: 387.3008 [*M*+H]^+^.

#### 2,6‐Bis‐[(2,2‐dimethylpent‐4‐enyl)aminomethyl]pyridine (28)

Was prepared according to the general procedure **B** from 2,6‐*bis*‐[(methylsulfonyloxy)methyl]pyridine (**22**; 1.29 g, 4.39 mmol) and 2,2‐dimethylpent‐4‐en‐1‐amine (**12**; 1.19 g, 10.53 mmol) yielding the title compound as a yellow oil (1.41 g, 4.28 mmol). ^1^H NMR (400 MHz, CDCl_3_): *δ*=7.58 (t, *J*=7.6 Hz, 1H), 7.18 (d, *J*=7.6 Hz, 2H), 5.85–5.74 (m, 2H), 5.03–4.98 (m, 4H), 3.88 (s, 4H), 2.39 (s, 4H), 2.03 (d, *J*=7.5 Hz, 4H), 0.91 (s, 12H) ppm; ^13^C NMR (101 MHz, CDCl_3_): *δ*=159.67, 136.80, 135.59, 120.33, 117.00, 60.16, 56.03, 44.82, 34.50, 25.62 ppm; HRMS (ESI): *m/z* calcd for C_21_H_36_N_3_^+^: 330.2904; found: 330.2901 [*M*+H]^+^.

#### General procedure C: Synthesis of *bis*‐*N*‐chloroamines (31–36)

Under a nitrogen atmosphere the crude unsaturated diamine was dissolved in anhydrous dichloromethane (10 mL/mmol of diamine) and freshly recrystallized *N*‐chlorosuccinimde (2.4 equiv) was added portion wise at 0 °C. The mixture was stirred at 0 °C for 30 min and for an additional 2–2.5 h at room temperature. The solvent was removed under reduced pressure and the crude product was purified by flash column chromatography.

#### 1,2‐*Bis*‐[*N*‐chloro(2,2‐dimethylpent‐4‐enyl)aminomethyl]benzene (31)

Was prepared according to the general procedure **C** from 1,2‐*Bis*‐[(2,2‐dimethylpent‐4‐enyl)aminomethyl]benzene (**23**; 1.48 g, 4.49 mmol) and purified by flash column chromatography (pentane/TBME 20 : 1), yielding the title compound as a pale yellow oil (1.35 g, 4.10 mmol, 66 % over 2 steps). ^1^H NMR (400 MHz, CDCl_3_): *δ*=7.39–7.35 (m, 2H), 7.32–7.28 (m, 2H), 5.78–5.66 (m, 2H), 5.02–4.95 (m, 4H), 4.28 (s, 4H), 2.94 (s, 4H), 2.03 (d, *J*=7.4 Hz, 4H), 0.92 (s, 12H) ppm; ^13^C NMR (101 MHz, CDCl_3_): *δ*=136.74, 135.22, 130.86, 127.92, 117.41, 73.59, 67.96, 45.19, 35.61, 26.03 ppm; HRMS (ESI): *m/z* calcd for C_22_H_35_Cl_2_N_2_^+^: 397.2172; found: 397.2170 [*M*+H]^+^.

#### 1,3‐*Bis*‐[*N*‐chloro(2,2‐dimethylpent‐4‐enyl)aminomethyl]benzene (32)

Was prepared according to the general procedure **C** from 1,3‐*Bis*‐[(2,2‐dimethylpent‐4‐enyl)aminomethyl]benzene (**24**; 1.47 g, 4.48 mmol) and purified by flash column chromatography (pentane/TBME 10 : 1), yielding the title compound as a colorless oil (1.63 g, 4.10 mmol, 80 % over 2 steps). ^1^H NMR (400 MHz, CDCl_3_): *δ*=7.38–7.30 (m, 4H), 5.83–5.71 (m, 2H), 5.05–4.97 (m, 4H), 4.13 (s, 4H), 2.93 (s, 4H), 2.08 (d, *J*=7.6 Hz, 4H), 0.95 (s, 12H) ppm; ^13^C NMR (50 MHz, CDCl_3_): *δ*=137.79, 135.33, 130.18, 128.81, 128.40, 117.43, 73.42, 70.49, 45.02, 35.75, 25.95 ppm; HRMS (ESI): *m/z* calcd for C_22_H_35_Cl_2_N_2_^+^: 397.2172; found: 397.2178 [*M*+H]^+^.

#### 5‐Methoxy‐1,3‐*bis*‐[*N*‐chloro(2,2‐dimethylpent‐4‐enyl)aminomethyl]benzene (34)

Was prepared according to the general procedure **C** from 5‐Methoxy‐1,3‐*bis*‐[(2,2‐dimethylpent‐4‐enyl)aminomethyl]benzene (**26**; 1.54 g, 4.28 mmol) and purified by flash column chromatography (pentane/TBME 20 : 1), yielding the title compound as a pale yellow oil (827 mg, 1.93 mmol, 44 % over 2 steps). ^1^H NMR (400 MHz, CDCl_3_): *δ*=6.94–6.92 (m, 1H), 6.88–6.86 (m, 2H), 5.82–5.71 (m, 2H), 5.03–5.02 (m, 2H), 5.01–4.97 (m, 2H), 4.09 (s, 4H), 3.82 (s, 3H), 2.91 (s, 4H), 2.07 (d, *J*=7.4 Hz, 4H), 0.94 (s, 12H) ppm; ^13^C NMR (101 MHz, CDCl_3_): *δ*=159.74, 139.19, 135.33, 122.34, 117.41, 114.18, 73.44, 70.50, 55.43, 45.07, 35.75, 25.98 ppm; HRMS (ESI): *m/z* calcd for C_23_H_36_Cl_2_N_2_ONa^+^: 449.2097; found: 449.2100 [*M*+Na]^+^.

#### Methyl 3,5‐*bis*‐[*N*‐chloro(2,2‐dimethylpent‐4‐enyl)‐aminomethyl]benzoate (35)

Was prepared according to the general procedure **C** from methyl 3,5‐*bis*‐[(2,2‐dimethylpent‐4‐enyl)aminomethyl]benzoate (**27**; 1.37 g, 3.55 mmol) and purified by flash column chromatography (pentane/TBME 10 : 1), yielding the title compound as a colorless oil (546 mg, 1.20 mmol, 33 % over 2 steps). ^1^H NMR (400 MHz, CDCl_3_): *δ*=7.95 (d, *J*=1.5 Hz, 2H), 7.62–7.60 (m, 1H), 5.04–5.03 (m, 2H), 5.00 (d, *J*=5.4 Hz, 2H), 4.15 (s, 4H), 3.93 (s, 3H), 2.94 (s, 4H), 2.08 (d, *J*=7.5 Hz, 4H), 0.95 (s, 12H) ppm; ^13^C NMR (101 MHz, CDCl_3_): *δ*=167.00, 138.31, 135.24, 134.59, 130.44, 129.90, 117.52, 73.71, 69.99, 52.35, 45.06, 35.83, 25.95 ppm; HRMS (ESI): *m/z* calcd for C_24_H_37_Cl_2_N_2_O_2_^+^:455.2227; found: 455.2231 [*M*+H]^+^.

#### 2,6‐*Bis*‐[*N*‐chloro(2,2‐dimethylpent‐4‐enyl)aminomethyl]pyridine (36)

Was prepared according to the general procedure **C** from 2,6‐*Bis*‐[(2,2‐dimethylpent‐4‐enyl)aminomethyl]pyridine (**28**; 1.31 g, 3.98 mmol) and purified by flash column chromatography (pentane/TBME 10 : 1), yielding the title compound as a colorless oil (284 mg, 0.71 mmol, 17 % over 2 steps). ^1^H NMR (400 MHz, CDCl_3_): *δ*=7.73 (t, *J*=7.7 Hz, 1H), 7.47 (d, *J*=7.7 Hz, 2H), 5.82–5.72 (m, 2H), 5.03–4.97 (m, 4H), 4.28 (s, 4H), 3.01 (s, 4H), 2.07 (d, *J*=7.5 Hz, 4H), 0.94 (s, 12H) ppm; ^13^C NMR (101 MHz, CDCl_3_): *δ*=156.85, 137.11, 135.25, 122.34, 117.51, 73.96, 71.93, 44.97, 35.85, 25.85 ppm; HRMS (ESI): *m/z* calcd for C_21_H_34_Cl_2_N_3_^+^: 398.2125; found: 398.2126 [*M*+H]^+^.

#### General procedure D: Synthesis of bis‐3‐chloropiperidines (1–6)

Under a nitrogen atmosphere the appropriate *bis*‐*N*‐chloroamine was dissolved in anhydrous chloroform (10 mL/mmol of *bis*‐*N*‐chloroamine) and tetrabutylammonium iodide (10 mol%) was added. The mixture was heated to 60 °C (oil bath temperature) for 2–2.5 h and the solvent was removed under reduced pressure. The product was purified by flash column chromatography. The resulting bis‐3‐chloropiperidines were obtained as an inseparable mixture of diastereomeres.

#### 1,2‐Bis‐[(3‐Chloro‐5,5‐dimethylpiperidin‐1‐yl)methyl]benzene (1)

Was prepared according to the general procedure **D** from 1,2‐*bis*‐[*N*‐chloro(2,2‐dimethylpent‐4‐enyl)aminomethyl]benzene (**31**; 1.34 g, 3.36 mmol) and purified by flash column chromatography (pentane/TBME 20 : 1), yielding the title compound as a pale yellow oil (1.07 g, 2.69 mmol, 80 %). ^1^H NMR (400 MHz, CDCl_3_): *δ*=7.32–7.26 (m, 2H), 7.24–7.19 (m, 2H), 4.09–3.98 (m, 2H), 3.74 (d, *J*=13.1 Hz, 1H), 3.59 (q, *J*=13.2 Hz, 2H), 3.43 (d, *J*=13.1 Hz, 1H), 3.13–3.03 (m, 2H), 2.42–2.32 (m, 2H), 2.00–1.89 (m, 4H), 1.83–1.75 (m, 2H), 1.36 (t, *J*=12.3 Hz, 2H), 1.04 (d, *J*=7.4 Hz, 6H), 0.91 (s, 6H) ppm; ^13^C NMR (101 MHz, CDCl_3_): *δ*=^13^C NMR (101 MHz, CDCl_3_): *δ*=137.46, 130.32, 127.08, 77.16, 65.59, 61.74, 60.04, 54.36, 48.49, 33.60, 29.45, 25.52 ppm; HRMS (ESI): *m/z* calcd for C_22_H_35_Cl_2_N_2_^+^:397.2172; found: 397.2175 [*M*+H]^+^; elemental analysis calcd (%) for C_22_H_34_Cl_2_N_2_: C 66.49; H 8.62; N 7.05; found: C 66.23; H 8.54; N 6.72.

#### 1,3‐Bis‐[(3‐chloro‐5,5‐dimethylpiperidin‐1‐yl)methyl]‐benzene (2)

Was prepared according to the general procedure **D** from 1,3‐*bis*‐[*N*‐chloro(2,2‐dimethylpent‐4‐enyl)aminomethyl]benzene (**32**; 950 mg, 2.39 mmol) and purified by flash column chromatography (pentane/TBME 10 : 1), yielding the title compound as a pale yellow oil (651 mg, 1.64 mmol, 69 %). ^1^H NMR (400 MHz, CDCl_3_): *δ*=7.27–7.15 (m, 4H), 4.16–4.06 (m, 2H), 3.58–3.43 (m, 4H), 3.20–3.12 (m, 2H), 2.43–2.35 (m, 2H), 2.04–1.92 (m, 4H), 1.76 (dd, *J*=10.9, 6.7 Hz, 2H), 1.35 (t, *J*=12.3 Hz, 2H), 1.06 (d, *J*=2.8 Hz, 6H), 0.89 (d, *J*=1.5 Hz, 6H) ppm; ^13^C NMR (101 MHz, CDCl_3_): *δ*=138.61, 129.15, 128.25, 127.58, 64.69, 62.43, 62.08, 54.36, 48.56, 33.53, 29.43, 25.27 ppm; HRMS (ESI): *m/z* calcd for C_22_H_35_Cl_2_N_2_^+^:397.2172; found: 397.2176 [*M*+H]^+^.

#### 5‐Methoxy‐1,3‐*bis*‐[(3‐Chloro‐5,5‐dimethylpiperidin‐1‐yl)methyl]benzene (4)

Was prepared according to the general procedure **D** from 5‐methoxy‐1,3‐*bis*‐[*N*‐chloro(2,2‐dimethylpent‐4‐enyl)aminomethyl]benzene (**34**; 1.31 g, 3.06 mmol) and purified by flash column chromatography (pentane/TBME 10 : 1), yielding the title compound as a pale orange oil (981 mg, 2.29 mmol, 75 %). ^1^H NMR (400 MHz, CDCl_3_): *δ*=6.84–6.80 (m, 1H), 6.80–6.75 (m, 2H), 4.17–4.06 (m, 2H), 3.80 (s, 3H), 3.56–3.37 (m, 4H), 3.20–3.11 (m, 2H), 2.43–2.34 (m, 2H), 2.05–1.91 (m, 4H), 1.78–1.70 (m, 2H), 1.35 (t, *J*=12.3 Hz, 2H), 1.07 (s, 6H), 0.89 (s, 6H) ppm; ^13^C NMR (101 MHz, CDCl_3_): *δ*=159.87, 140.21, 121.37, 112.77, 77.16, 64.64, 62.33, 55.34, 54.36, 48.51, 33.52, 29.42, 25.27 ppm; HRMS (ESI): *m/z* calcd for C_23_H_37_Cl_2_N_2_O^+^: 427.2278; found: 427.2281 [*M*+H]^+^; elemental analysis calcd (%) for C_23_H_36_Cl_2_N_2_O: C 64.63; H 8.49; N 6.55; found: C 64.96; H 8.29; N 6.62.

#### Methyl 3,5‐*bis*‐[(3‐chloro‐5,5‐dimethylpiperidin‐1‐yl)methyl]‐benzoate (5)

Was prepared according to the general procedure **D** from methyl 3,5‐*bis*‐[*N*‐chloro(2,2‐dimethylpent‐4‐enyl)aminomethyl]benzoate (**35**; 514 mg, 1.13 mmol) and purified by flash column chromatography (pentane/TBME 10 : 1), yielding the title compound as a pale yellow oil (447 mg, 0.98 mmol, 86 %). ^1^H NMR (400 MHz, CDCl_3_): *δ*=7.87–7.82 (m, 2H), 7.48 (d, *J*=7.8 Hz, 1H), 4.10 (s, 2H), 3.92 (s, 3H), 3.61–3.46 (m, 4H), 3.18–3.09 (m, 2H), 2.36 (t, *J*=9.9 Hz, 2H), 2.06–1.92 (m, 4H), 1.80–1.73 (m, 2H), 1.35 (t, *J*=12.3 Hz, 2H), 1.06 (d, *J*=3.3 Hz, 6H), 0.89 (s, 6H) ppm; ^13^C NMR (101 MHz, CDCl_3_): *δ*=167.33, 139.19, 133.65, 130.36, 128.82, 77.16, 64.68, 62.01, 54.18, 52.27, 48.45, 33.53, 29.40, 25.25 ppm; HRMS (ESI): *m/z* calcd for C_24_H_37_Cl_2_N_2_O_2_^+^: 455.2227; found: 455.2228 [*M*+H]^+^.

#### 2,6‐Bis‐[(3‐chloro‐5,5‐dimethylpiperidin‐1‐yl)methyl]‐pyridine (6)

Was prepared according to the general procedure **D** from 2,6‐*Bis*‐[*N*‐chloro(2,2‐dimethylpent‐4‐enyl)aminomethyl]pyridine (**36**; 240 mg, 0.60 mmol) and purified by flash column chromatography (pentane/TBME 3 : 1), yielding the title compound as a pale yellow oil (183 mg, 0.46 mmol, 76 %). ^1^H NMR (400 MHz, CDCl_3_): *δ*=7.64 (t, *J*=7.7 Hz, 1H), 7.34 (d, *J*=7.1 Hz, 2H), 4.19–4.10 (m, 2H), 3.76–3.59 (m, 4H), 3.19 (d, *J*=8.5 Hz, 2H), 2.40 (d, *J*=10.9 Hz, 2H), 2.14 (t, *J*=10.6 Hz, 2H), 1.98–1.86 (m, 4H), 1.37 (t, *J*=12.3 Hz, 2H), 1.09 (s, 6H), 0.90 (s, 6H) ppm; ^13^C NMR (101 MHz, CDCl_3_): *δ*=158.30, 137.07, 121.00, 64.79, 63.95, 62.18, 54.02, 48.26, 33.53, 29.39, 25.30 ppm; HRMS (ESI): *m/z* calcd for C_21_H_34_Cl_2_N_3_^+^: 398.2125; found: 398.2124 [*M*+H]^+^.

#### 1,3,5‐Tris‐[(2,2‐dimethylpent‐4‐enyl)aminomethyl]benzene (42)

Under a nitrogen atmosphere 1,3,5‐*tris*(bromomethyl)benzene (**41**; 1.00 g, 2.81 mmol) was dissolved in anhydrous dichloromethane (60 mL) and 2,2‐dimethylpent‐4‐en‐1amine (**12**; 1.90 g, 16.78 mmol) was added dropwise at 0 °C. The mixture was stirred at room temperature for 68 h and was then quenched by the addition of distilled water (50 mL). The layers were separated, the organic phase washed with brine and was then dried over MgSO_4_. The solvent was removed under reduced pressure and the crude product was obtained as a yellow oil, which was used in the next step without further purification (1.17 g, 2.59 mmol). ^1^H NMR (400 MHz, CDCl_3_): *δ*=7.16 (s, 3H), 5.84–5.74 (m, 3H), 5.00 (d, *J*=13.5 Hz, 6H), 3.78 (s, 6H), 2.37 (s, 6H), 2.02 (d, *J*=7.5 Hz, 6H), 0.89 (s, 18H) ppm; ^13^C NMR (101 MHz, CDCl_3_): *δ*=141.07, 135.72, 126.21, 116.89, 59.88, 54.81, 44.78, 34.50, 25.67 ppm; HRMS (ESI): *m/z* calcd for C_30_H_52_N_3_^+^: 454.4156; found: 454.4154 [*M*+H]^+^.

#### 1,3,5‐*Tris*‐[*N*‐chloro‐(2,2‐dimethylpent‐4‐enyl)aminomethyl]benzene (43)

Under a nitrogen atmosphere 1,3,5‐*tris*‐[(2,2‐dimethylpent‐4‐enyl)aminomethyl]benzene (**42**; 1.13 g, 2.49 mmol) was dissolved in anhydrous dichloromethane (80 mL) and freshly recrystallized *N*‐chlorosuccinimde (1.10 g, 8.22 mmol) was added portion wise at 0 °C. The mixture was stirred at 0 °C for 30 min and for an additional 2 h at room temperature. The solvent was removed under reduced pressure and the crude product was purified by flash column chromatography (pentane/TBME 10 : 1). The product was obtained as a colorless oil (1.09 g, 1.95 mmol, 75 % over 2 steps). ^1^H NMR (400 MHz, CDCl_3_): *δ*=7.32–7.27 (m, 3H), 5.81–5.71 (m, 3H), 5.03–4.97 (m, 6H), 4.12 (s, 6H), 2.92 (s, 6H), 2.06 (d, *J*=7.5 Hz, 6H), 0.94 (s, 18H) ppm; ^13^C NMR (101 MHz, CDCl_3_): *δ*=137.84, 135.34, 129.73, 117.43, 77.16, 73.37, 70.39, 45.06, 35.75, 25.96 ppm; HRMS (ESI): *m/z* calcd for C_30_H_48_Cl_3_N_3_Na^+^: 578.2806; found: 578.2806 [*M*+H]^+^.

#### 1,3,5‐Tris‐[(3‐chloro‐5,5‐dimethylpiperidin‐1‐yl)methyl]‐benzene (8)

Under a nitrogen atmosphere 1,3,5‐*Tris*‐[*N*‐chloro‐(2,2‐dimethylpent‐4‐enyl)aminomethyl]benzene (**43**; 1.03 g, 1.85 mmol) was dissolved in anhydrous chloroform (20 mL) and tetrabutylammonium iodide (81 mg, 14 mol%) was added. The mixture was heated to reflux for 4 h and the solvent was removed under reduced pressure. The product was purified by flash column chromatography (pentane/TBME 10 : 1) and was obtained as a pale yellow oil (0.70 g, 1.26 mmol, 68 %). ^1^H NMR (400 MHz, CDCl_3_): *δ*=7.14–7.06 (m, 3H), 4.14–4.06 (m, 3H), 3.56–3.40 (m, 6H), 3.19–3.11 (m, 3H), 2.41–2.33 (m, 3H), 2.03–1.91 (m, 6H), 1.74 (dt, *J*=11.0, 5.4 Hz, 3H), 1.34 (t, *J*=12.3 Hz, 3H), 1.05 (t, *J*=3.1 Hz, 9H), 0.89 (s, 9H) ppm; ^13^C NMR (101 MHz, CDCl_3_): *δ*=138.51, 128.02, 64.66, 62.41, 62.10, 54.39, 48.53, 33.53, 29.44, 25.31 ppm; HRMS (ESI): *m/z* calcd for C_30_H_49_Cl_3_N_3_^+^: 556.2987; found: 556.2988 [*M*+H]^+^; elemental analysis calcd (%) for C_30_H_48_Cl_3_N_3_: C 64.68; H 8.69; N 7.44; found: C 64.58; H 8.52; N 7.44.

## Conflict of interest

The authors declare no conflict of interest.

## Supporting information

As a service to our authors and readers, this journal provides supporting information supplied by the authors. Such materials are peer reviewed and may be re‐organized for online delivery, but are not copy‐edited or typeset. Technical support issues arising from supporting information (other than missing files) should be addressed to the authors.

Supplementary

## References

[1a] N. Saijo , Cancer Res. 2012, 44, 1–10; 10.4143/crt.2012.44.1.1 PMC3322195 22500155

[1b] M. Huang , A. J. Shen , J. Ding , M. Y. Geng , Trends Pharmacol. Sci. 2014, 35, 41–50. 24361003 10.1016/j.tips.2013.11.004

[2] B. A. Chabner , T. G. Roberts Jr. , Nat. Rev. Cancer 2005, 5, 65–72. 15630416 10.1038/nrc1529

[3] R. K. Singh , S. Kumar , D. N. Prasad , T. R. Bhardwaj , Eur. J. Med. Chem. 2018, 151, 401–433. 29649739 10.1016/j.ejmech.2018.04.001

[4a] I. Zuravka , R. Roesmann , A. Sosic , R. Gottlich , B. Gatto , Bioorg. Med. Chem. 2015, 23, 1241–1250; 25693786 10.1016/j.bmc.2015.01.050

[4b] I. Zuravka , R. Roesmann , A. Sosic , W. Wende , A. Pingoud , B. Gatto , R. Gottlich , ChemMedChem 2014, 9, 2178–2185; 24616300 10.1002/cmdc.201400034

[4c] I. Zuravka , A. Sosic , B. Gatto , R. Gottlich , Bioorg. Med. Chem. Lett. 2015, 25, 4606–4609. 26342869 10.1016/j.bmcl.2015.08.042

[5] A. Sosic , I. Zuravka , N. K. Schmitt , A. Miola , R. Gottlich , D. Fabris , B. Gatto , ChemMedChem 2017, 12, 1471–1479. 28724198 10.1002/cmdc.201700368 PMC7054790

[6a] A. Polavarapu , J. A. Stillabower , S. G. Stubblefield , W. M. Taylor , M. H. Baik , J. Org. Chem. 2012, 77, 5914–5921; 22681226 10.1021/jo300351g

[6b] R. B. Silverman, M. W. Holladay, *The Organic Chemistry of Drug Design and Drug Action*, 3rd ed., Elsevier, Amsterdam, **2014**.

[7] K. C. Brannock , J. Am. Chem. Soc. 1959, 81, 3379–3383.

[8] M. Noack , R. Göttlich , Eur. J. Org. Chem. 2002, 2002, 3171–3178.

[9] C. Carraro , A. Francke , A. Sosic , F. Kohl , T. Helbing , M. De Franco , D. Fabris , R. Gottlich , B. Gatto , ACS Med. Chem. Lett. 2019, 10, 552–557. 30996795 10.1021/acsmedchemlett.8b00580 PMC6466835

[10] L. P. Hammett , J. Am. Chem. Soc. 1937, 59, 96–103.

[11] C. J. O′Connor , W. A. Denny , J. Y. Fan , G. L. Gravatt , B. A. Grigor , D. J. Mclennan , J. Chem. Soc. Perkin Trans. 2 1991, 1933–1939.

[12] B. Varghese , S. Kothari , K. K. Banerji , Int. J. Chem. Kinet. 1999, 31, 245–252.

[13] C. Bolzati , D. Carta , V. Gandin , C. Marzano , N. Morellato , N. Salvarese , M. Cantore , N. A. Colabufo , J. Biol. Inorg. Chem. 2013, 18, 523–538. 23543234 10.1007/s00775-013-0997-1

[14] D. W. Fairbairn , P. L. Olive , K. L. Oneill , Mutat. Res. 1995, 339, 37–59. 7877644 10.1016/0165-1110(94)00013-3

[15] R. Edmondson , J. J. Broglie , A. F. Adcock , L. Yang , Assay Drug Dev. Technol. 2014, 12, 207–218. 24831787 10.1089/adt.2014.573 PMC4026212

[16] V. Gandin , C. Ceresa , G. Esposito , S. Indraccolo , M. Porchia , F. Tisato , C. Santini , M. Pellei , C. Marzano , Sci. Rep. 2017, 7, 13936 10.1038/s41598-017-13698-1. 29066771 10.1038/s41598-017-13698-1 PMC5655689

[17] E. M. Tacconi , S. Badie , G. De Gregoriis , T. Reislander , X. Lai , M. Porru , C. Folio , J. Moore , A. Kopp , J. Baguna Torres , D. Sneddon , M. Green , S. Dedic , J. W. Lee , A. S. Batra , O. M. Rueda , A. Bruna , C. Leonetti , C. Caldas , B. Cornelissen , L. Brino , A. Ryan , A. Biroccio , M. Tarsounas , EMBO Mol. Med. 2019, 11, e9982. 31273933 10.15252/emmm.201809982 PMC6609913

[18a] D. Montagner , V. Gandin , C. Marzano , A. Erxleben , J. Inorg. Biochem. 2015, 145, 101–107; 25660211 10.1016/j.jinorgbio.2015.01.013

[18b] M. C. Alley , D. A. Scudiero , A. Monks , M. L. Hursey , M. J. Czerwinski , D. L. Fine , B. J. Abbott , J. G. Mayo , R. H. Shoemaker , M. R. Boyd , Cancer Res. 1988, 48, 589–601. 3335022

[19] F. Arnesano , M. Losacco , G. Natile , Eur. J. Inorg. Chem. 2013, 2701–2711. 10.1002/ejic.201300001.

[20] C. Marzano , S. M. Sbovata , V. Gandin , D. Colavito , E. Del Giudice , R. A. Michelin , A. Venzo , R. Seraglia , F. Benetollo , M. Schiavon , R. Bertani , J. Med. Chem. 2010, 53, 6210–6227. 20681543 10.1021/jm1006534

