# Supplementary material for: Aromatic Linkers Unleash the Antiproliferative Potential of 3‐Chloropiperidines Against Pancreatic Cancer Cells
Source: ChemMedChem. 2020 Sep 15;15(21):2040–51. doi: 10.1002/cmdc.202000457 (PMC7692949; doi:10.1002/cmdc.202000457)
Supplement: Supplementary file 1 — Supplementary [file CMDC-15-2040-s001.pdf]

ChemMedChem

Supporting Information

Aromatic Linkers Unleash the Antiproliferative Potential of 3-Chloropiperidines Against Pancreatic Cancer Cells

Tim Helbing[+], Caterina Carraro[+], Alexander Francke, Alice Sosic, Michele De Franco, Valentina Gandin, Richard Göttlich,* and Barbara Gatto*

Supporting Information

Table of contents

Supporting figures and tables **S2**

Synthetic procedures of the precursors 10-13, 18-22 and 40-41 **S4**

References **S9**

Copies of ^{1}H and ^{13}C NMR spectra of the B-CePs 1, 2, 4, 5, 6 and the Tri-CeP 8 **S9**

Supporting figures and tables

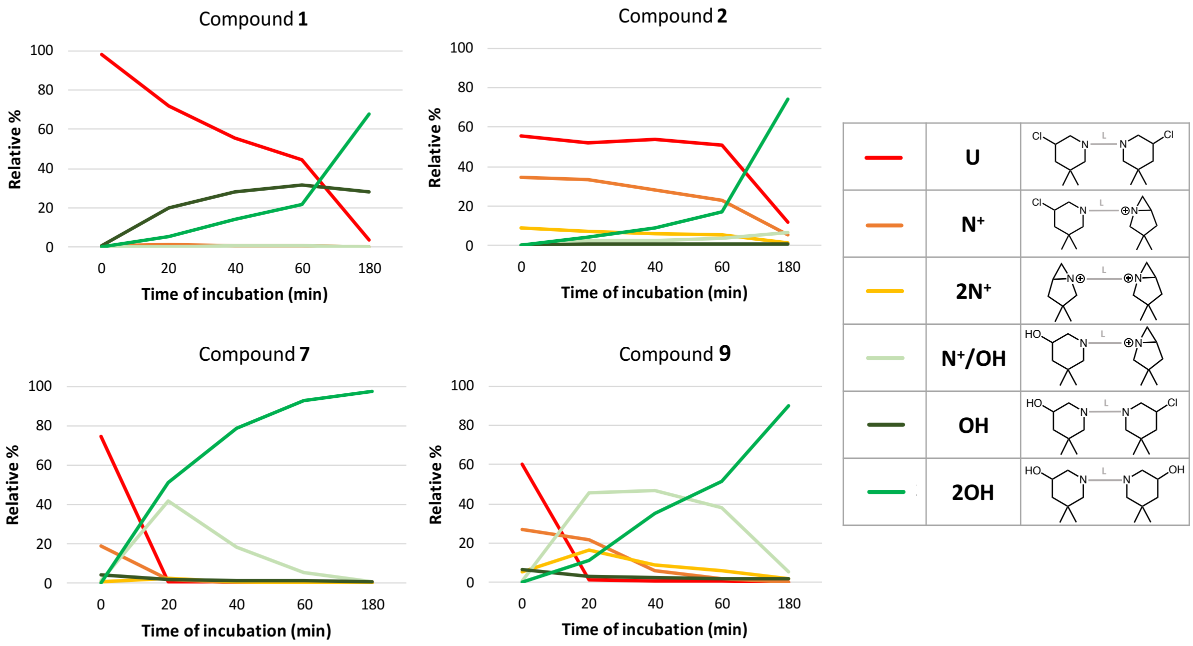

Figure S1. Time-followed formation of B-CePs reaction species at 37 °C in water detected by ESI-MS. Graphs report the relative percentages of reaction species (U = unreacted compound, N^+ = aziridinium ion, $2N^+$ = double aziridinium ion, OH = monohydroxylated, N^+/OH = monohydroxylated/aziridinium ion, 2OH = dihydroxylated) resulting from the incubation of compounds **1**, **2**, **7**, **9** up to 3 h in water at 37 °C and detected by ESI-MS.

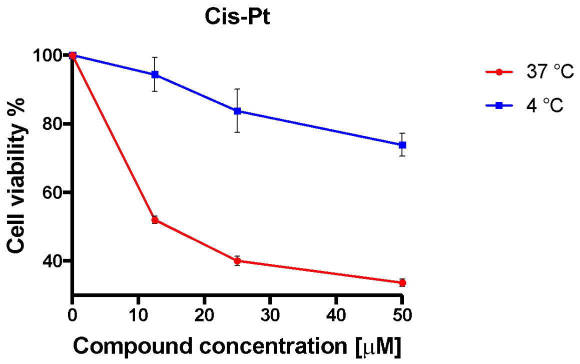

Figure S2. Cell viability curve for BxPC-3 cells upon treatment at 37 °c vs 4 °C with cisplatin (Cis-Pt). BxPC-3 cell viability upon exposure to increasing concentrations of Cis-Pt for 5 h at 37 °C (red curve) or 4 °C (blue curve), followed by a gentle rinse of wells with PBS and addition of fresh RPMI medium. The MTT assay was performed after 72 h.

Table S1. Plasmid DNA cleavage of test compounds.

Compound	Cleavage EC_{50}s [µM]
**1**	89.2±1.4
**2**	1.6±0.2
**3**	2.0±0.1
**4**	3.1±0.8
**5**	3.8±1.0
**6**	3.1±0.1
**7**	0.8±0.1
**8**	13.1±1.4
**9**	0.9±0.1

The supercoiled pBR322 plasmid was incubated with increasing concentrations of compounds at 37 °C for 3 h in BPE buffer. EC_{50} values were calculated by comparing the intensity of the supercoiled species band to the negative control as a function of compound concentration. Average EC_{50}s and standard deviations result from two independent experiments.

Synthetic procedures of the precursors 10-13, 16-20 and 34-35

2,2-Dimethylpent-4-enal (10)

Freshly destilled isobutyraldehyde (108.0 g, 1.5 mol) and allyl alcohol (58.0 g, 1.0 mol) were dissolved in *p*-cymene (170 mL) and *p*-toluenesulfonic acid (0.25 g) were added. The mixture was heated to reflux for 32 h using a Dean-Stark apparatus, until no more water was separated and a sump temperature of about 140 °C was reached. The product was separated by vacuum distillation (76 °C at 200 mbar) through a 50 cm Vigurex column and was obtained as a colourless liquid (81.4 g, 0.73 mol, 73%). ^{1}H NMR (400 MHz, $CDCl_3$): δ = 9.45 (s, 1H), 5.74 – 5.61 (m, 1H), 5.07 – 5.01 (m, 2H), 2.19 (d, J = 7.4 Hz, 2H), 1.03 (s, 6H) ppm; ^{13}C NMR (101 MHz, $CDCl_3$): δ = 205.91, 133.24, 118.53, 41.53, 21.24 ppm. These data are consistent with published data.[1]

2,2-Dimethylpent-4-enal oxime (11)

To a solution of hydroxylammonium chloride (15.05 g, 216.78 mmol) in distilled water (20 mL) a solution of sodium hydroxide (7.23 g, 180.65 mmol) in distilled water/ethanol (1:3, 80 mL) was added. Afterwards a solution of 2,2-dimethylpent-4-enal **(10)** (20.26 g, 180.65 mmol) in ethanol (300 mL) was added and the mixture was left standing overnight. Most of the ethanol was removed in vaccuo and TBME (300 mL) was added. The layers were separated and the aqueous phase was extracted with TBME (3x 100 mL). The combined organic extracts were dried over $MgSO_4$ and the solvent was removed under reduced pressure. The product was purified by vacuum distillation (96 °C at 40 mbar) and was obtained as a colourless liquid (19.35 g, 152.18 mmol, 84%). ^{1}H NMR (400 MHz, $CDCl_3$): δ = 7.34 (s, 1H), 5.81 – 5.70 (m, 1H), 5.10 – 5.02 (m, 2H), 2.15 (d, J = 7.4 Hz, 2H), 1.09 (s, 6H) ppm; ^{13}C NMR (101 MHz, $CDCl_3$): δ = 158.65, 134.01, 118.25, 45.37, 36.75, 25.14 ppm; HRMS (ESI): *m/z* calcd for $C_7H_{13}NNaO^+$: 150.0889; found: 150.0887 $[M+Na]^+$. These data are consistent with published data.[2]

2,2-Dimethylpent-4-en-1-amine (12)

Under a nitrogen atmosphere lithium aluminium hydride (5.83 g, 153.54 mmol) was suspended in anhydrous diethyl ether (150 mL) and a solution of 2,2-dimethylpent-4-enal oxime **(11)** (13.19 g, 103.75 mmol) in anhydrous diethyl ether (50 mL) was added dropwise at 0 °C. The mixture was stirred at room temperature for 18 h and afterwards quenched by the careful addition of distilled water (6 mL), followed by 15% NaOH solution (6 mL) and finally distilled water (20 mL) at 0 °C. The

mixture was stirred for an additional 30 min at 0 °C and then filtered. The precipitate was again suspended in diethyl ether (100 mL), heated to reflux for 30 min and filtered afterwards. The combined organic filtrates were dried over $MgSO_4$ and the solvent was removed under reduced pressure. The product was purified by vacuum distillation (65 °C at 80 mbar) and was obtained as a colourless liquid (5.86 g, 51.77 mmol, 50%). ^1H NMR (400 MHz, $CDCl_3$): δ = 5.82 – 5.71 (m, 1H), 5.01 – 4.95 (m, 2H), 2.40 (s, 2H), 1.92 (d, J = 8.5 Hz, 2H), 1.10 (b, 2H), 0.80 (s, 6H) ppm; ^{13}C NMR (101 MHz, $CDCl_3$): δ = 135.38, 116.95, 52.72, 44.06, 34.95, 24.65 ppm; HRMS (ESI): *m/z* calcd for $C_8H_{15}N^+$: 114.1277; found: 114.1282 $[M+H]^+$. These data are consistent with published data.[2]

1,2-*Bis*-(aminomethyl)benzene (13)

Under a nitrogen atmosphere 1,2-*Bis*(bromomethyl)benzene (5.28 g, 20.00 mmol) was dissolved in anhydrous dimethylformamide (50 mL) and sodium azide (5.20 g, 80.00 mmol) was added. The mixture was heated to 80 °C for 3 h and then quenched by the addition of distilled water (40 mL). The aqueous phase was extracted with ethyl acetate (3x 50 mL), the combined organic extracts were washed with brine and dried over $MgSO_4$. The mixture was then concentrated under reduced pressure until approximately 10 mL remained and tetrahydrofuran (180 mL) was added. Afterwards triphenylphosphine (15.74 g, 60.01 mmol) was added portion wise and the mixture was stirred for 30 min at room temperature. After the addition of distilled water (10 mL) the mixture was stirred for another 3 h at room temperature. The solvent was removed under reduced pressure and ethyl acetate (50 mL) was added. The organic phase was extracted with 1 M HCl solution (3x 50 mL) and the pH was adjusted to 12 by addition of solid KOH. The combined aqueous phases were then extracted with dichloromethane (3x 70 mL). The resulting combined organic extracts were dried over $MgSO_4$ and the solvent was removed under reduced pressure. The product was purified by vacuum distillation (105 °C at 0.42 mbar) and was obtained as a colourless oil (1.31 g, 9.62 mmol, 48%). ^1H NMR (400 MHz, $CDCl_3$): δ = 7.44 – 7.40 (m, 2H), 7.39 – 7.35 (m, 2H), 4.03 (s, 4H), 2.21 (b, 4H) ppm; ^{13}C NMR (101 MHz, $CDCl_3$): δ = 140.81, 128.87, 127.59, 44.25 ppm; HRMS (ESI): *m/z* calcd for $C_8H_{12}N_2^+$: 137.1073; found: 137.1073 $[M+H]^+$. These data are consistent with published data.[3]

Dimethyl 5-methoxyisophthalate (18)

Under a nitrogen atmosphere dimethyl 5-hydroxyisophthalate (6.30 g, 30.00 mmol) was dissolved in anhydrous dimethylformamide (50 mL) and K_2CO_3 (12.44 g, 90.00 mmol) was added. The mixture was stirred at room temperature for 15 min. Afterwards methyl iodide (6.38 g, 45.00 mmol) was added drop wise and the mixture was stirred at room temperature for 18 h. The reaction was quenched by

the addition of distilled water (60 mL) and then ethyl acetate (250 mL) was added. The layers were separated and the organic phase was washed with distilled water (5x 100 mL). The organic phase was then dried over $MgSO_4$ and the solvent was removed under reduced pressure. The product was dried over P_2O_5 in a desiccator and was obtained as a white solid (6.02 g, 26.83 mmol, 89%). ^1H NMR (400 MHz, $CDCl_3$) δ = 8.26 (t, *J* = 1.4 Hz, 1H), 7.73 (d, *J* = 1.4 Hz, 2H), 3.93 (s, 6H), 3.88 (s, 3H) ppm; ^{13}C NMR (101 MHz, $CDCl_3$) δ = 166.29, 159.81, 131.91, 123.09, 119.43, 55.94, 52.56 ppm; HRMS (ESI): *m/z* calcd for $C_{11}H_{12}O_5Na^+$: 247.0577; found: 247.0577 $[M+Na]^+$. These data are consistent with published data.[4]

5-Methoxy-1,3-benzenedimethanol (19)

Under a nitrogen atmosphere lithium aluminium hydride (1.40 g, 36.88 mmol) was suspended in anhydrous tetrahydrofuran (40 mL) and a solution of dimethyl 5-methoxyisophthalate **(18)** (3.06 g, 13.66 mmol) in anhydrous tetrahydrofuran (20 mL) was added dropwise at 0 °C. The mixture was stirred at 0 °C for 15 min and at room temperature for 16 h. The reaction was then quenched by the careful addition of distilled water (10 mL) and afterwards 10% H_2SO_4 (about 40 mL) was added, until the precipitated aluminium oxide was dissolved completely. The layers were separated and the aqueous phase was extracted with ethyl acetate (3x 50 mL). The combined organic extracts were washed with brine and dried over $MgSO_4$. The solvent was removed under reduced pressure and the product was obtained as white solid (2.19 g, 13.00 mmol, 95%). ^1H NMR (400 MHz, $CDCl_3$) δ = 6.92 (s, 1H), 6.82 (s, 2H), 4.64 (s, 4H), 3.81 (s, 3H), 1.98 (s, 2H) ppm; ^{13}C NMR (101 MHz, $CDCl_3$) δ = 160.08, 142.89, 117.67, 111.66, 77.16, 65.07, 55.44 ppm; HRMS (ESI): *m/z* calcd for $C_9H_{12}O_3Na^+$: 191.0682; found: 191.0682 $[M+Na]^+$. These data are consistent with published data.[4]

5-Methoxy-1,3-*bis*-[(methylsulfonyloxy)methyl]benzene (20)

Under a nitrogen atmosphere 5-methoxy-1,3-benzenedimethanol **(19)** (1.98 g, 11.77 mmol) was dissolved in anhydrous dichloromethane (60 mL) and anhydrous triethylamine (4.89 mL, 35.31 mmol) was added dropwise. The mixture was stirred at room temperature for 5 min and then cooled to - 5 °C. Afterwards freshly distilled methanesulfonyl chloride (2.28 mL, 29.42 mmol) was added dropwise and the mixture was stirred at -5 °C for 5 h. The reaction was then quenched by the addition of distilled water (20 mL) and the layers were separated. The aqueous phase was extracted with dichloromethane (3x 30 mL), the combined organic extracts were washed saturated NH_4Cl solution and dried over $MgSO_4$. The solvent was removed under reduced pressure and the crude product was purified by flash chromatography (ethyl acetate/cyclohexane 2:1). The product was

obtained as a colourless oil (2.66 g, 8.20 mmol, 70%). ^{1}H NMR (400 MHz, $CDCl_3$) δ = 7.03 (s, 1H), 6.96 (s, 2H), 5.21 (s, 4H), 3.84 (s, 3H), 2.98 (s, 6H) ppm; ^{13}C NMR (101 MHz, $CDCl_3$) δ = 160.47, 135.88, 120.75, 115.02, 70.68, 55.71, 38.45 ppm; HRMS (ESI): *m/z* calcd for $C_{11}H_{16}O_7S_2Na^+$: 347.0229; found: 347.0228 $[M+Na]^+$.

Methyl 3,5-*bis*-(bromomethyl)benzoate (21)

Methyl 3,5-dimethylbenzoate (14.58 g, 88.86 mmol) was dissolved in carbon tetrachloride (150 mL) and *N*-bromosuccinimide (34.85 g, 195.80 mmol) was added. Afterwards nitrogen was bubbled through the suspension for 10 min. The mixture was heated to reflux and benzoyl peroxide (1.90 g, 7.83 mmol) was added portion wise during heating. The mixture was heated to reflux for another 4 h and then filtered. The filter cake was washed with carbon tetrachloride (30 mL) and the combined organic filtrates were washed with distilled water as well as brine. The organic phase was dried over $MgSO_4$ and the solvent was removed under reduced pressure. The resulting oil was dissolved in hot hexane/ethyl acetate (10:1) and crystallized at 7 °C. The resulting solid was then recrystallized from hexane/ethyl acetate and the product was obtained as a white solid (11.25 g, 34.95 mmol, 39%). ^{1}H NMR (400 MHz, $CDCl_3$) δ = 8.00 (s, 2H), 7.62 (s, 1H), 4.50 (s, 4H), 3.93 (s, 3H) ppm; ^{13}C NMR (101 MHz, $CDCl_3$) δ = 165.92, 138.96, 133.84, 131.43, 130.03, 52.42, 31.84 ppm; HRMS (ESI): *m/z* calcd for $C_{10}H_{10}Br_2O_2Na^+$: 344.8919; found: 344.8917 $[M+Na]^+$. These data are consistent with published data.[5]

2,6-*Bis*-[(methylsulfonyloxy)methyl]pyridin (22)

Under a nitrogen atmosphere 2,6-pyridindimethanol (2.00 g, 14.37 mmol) was dissolved in anhydrous dichloromethane (70 mL) and anhydrous triethylamine (5.96 mL, 43.11 mmol) was added dropwise. The mixture was stirred at room temperature for 5 min and then cooled to -5 °C. Afterwards freshly distilled methanesulfonyl chloride (2.78 mL, 35.92 mmol) was added dropwise and the mixture was stirred at -5 °C for 3 h. The reaction was then quenched by the addition of distilled water (25 mL) and the layers were separated. The aqueous phase was extracted with dichloromethane (3x 30 mL), the combined organic extracts were washed saturated NH_4Cl solution and dried over $MgSO_4$. The solvent was removed under reduced pressure and the crude product was purified by flash chromatography (ethyl acetate/cyclohexane 3:1). The product was obtained as a white solid (3.36 g, 11.37 mmol, 79%). ^{1}H NMR (400 MHz, $CDCl_3$) δ = 7.83 (t, *J* = 7.8 Hz, 1H), 7.47 (d, *J* = 7.8 Hz, 2H), 5.31 (s, 4H), 3.10 (s, 6H) ppm; ^{13}C NMR (101 MHz, $CDCl_3$) δ = 153.90,

138.44, 122.35, 77.16, 71.12, 38.23 ppm; HRMS (ESI): *m/z* calcd for $C_9H_{13}O_6S_2Na^+$: 318.0081; found: 318.0078 $[M+Na]^+$. These data are consistent with published data.[6]

1,3,5-*Tris*-(hydroxymethyl)benzene (40)

Under a nitrogen atmosphere lithium aluminium hydride (8.90 g, 234.52 mmol) was suspended in anhydrous tetrahydrofuran (150 mL) and trimethyl 1,3,5-benzenetricarboxylate **(39)** (12.64 g, 50.10 mmol) was added portion wise at 0 °C. The mixture was heated to reflux for 30 min, was then stirred for 16 h at room temperature and was heated to reflux for another 2 h. The reactions was then quenched by the careful addition of distilled water (9 mL), 20% NaOH solution (9 mL) and finally distilled water (9 mL) at 0 °C. The mixture was stirred at room temperature for 1 h and was then filtered. The filter cake was again suspended in tetrahydrofuran (100 mL), heated to reflux for another 2 h and filtered afterwards. The combined filtrates were dried over $MgSO_4$ and the solvent was removed under reduced pressure. The product was obtained as a white solid (6.98 g, 41.51 mmol, 83%). ^{1}H NMR (400 MHz, D_2O) δ = 7.31 (s, 3H), 4.65 (s, 6H) ppm; ^{13}C NMR (101 MHz, D_2O) δ = 141.05, 125.56, 63.68 ppm; HRMS (ESI): *m/z* calcd for $C_9H_{12}O_3Na^+$: 191.0678; found: 191.0681 $[M+Na]^+$. These data are consistent with published data.[7]

1,3,5-*Tris*-(bromomethyl)benzene (41)

Under a nitrogen atmosphere 1,3,5-*tris*(hydroxymethyl)benzene **(40)** (2.70 g, 16.10 mmol) was dissolved in anhydrous diethyl ether (100 mL) and phosphorous tribromide (6.12 mL, 64.40 mmol) was added dropwise at 0 °C. The solution was stirred at 0 °C for 2 h and for an additional 22 h at room temperature. The mixture was poured on ice and the layers were separated. The aqueous phase was extracted with diethyl ether (3x 100 mL) and the combined organic extracts were dried over $MgSO_4$. The solvent was removed under reduced pressure and the crude product was purified by flash chromatography (dichloromethane/pentane 10:1). The product was obtained as a white solid (4.28 g, 11.99 mmol, 75%). ^{1}H NMR (400 MHz, $CDCl_3$) δ = 7.35 (s, 3H), 4.45 (s, 6H) ppm; ^{13}C NMR (101 MHz, $CDCl_3$) δ = 139.18, 129.71, 32.34 ppm; HRMS (ESI): *m/z* calcd for $C_9H_9Br_3Na^+$: 378.8126; found: 378.8126 $[M+Na]^+$. These data are consistent with published data.[7]

References

[1] K. C. Brannock, *J Am Chem Soc* **1959**, *81*, 3379.

[2] M. Noack, R. Göttlich, *Eur J Org Chem* **2002**, *2002*, 3171.

[3] N. Gigant, E. Claveau, P. Bouyssou, I. Gillaizeau, *Org Lett* **2012**, *14*, 844.

[4] H. Fan, H. Sun, X. Peng, *Chemistry* **2018**, *24*, 7671.

[5] T. Routasalo, J. Helaja, J. Kavakka, A. M. P. Koskinen, *Eur J Org Chem* **2008**, *2008*, 3190.

[6] A. J. Wessel, J. W. Schultz, F. Tang, H. Duan, L. M. Mirica, *Org Biomol Chem* **2017**, *15*, 9923.

[7] L. Gil, Y. Han, E. E. Opas, G. A. Rodan, R. Ruel, J.G. Seedor, P. C. Tyler, R. N. Young, *Bioorg Med Chem* **1999**, *7*, 901.

Copies of ^{1}H and ^{13}C NMR spectra of the B-CePs 1, 2, 4, 5, 6 and the Tri-CeP 8

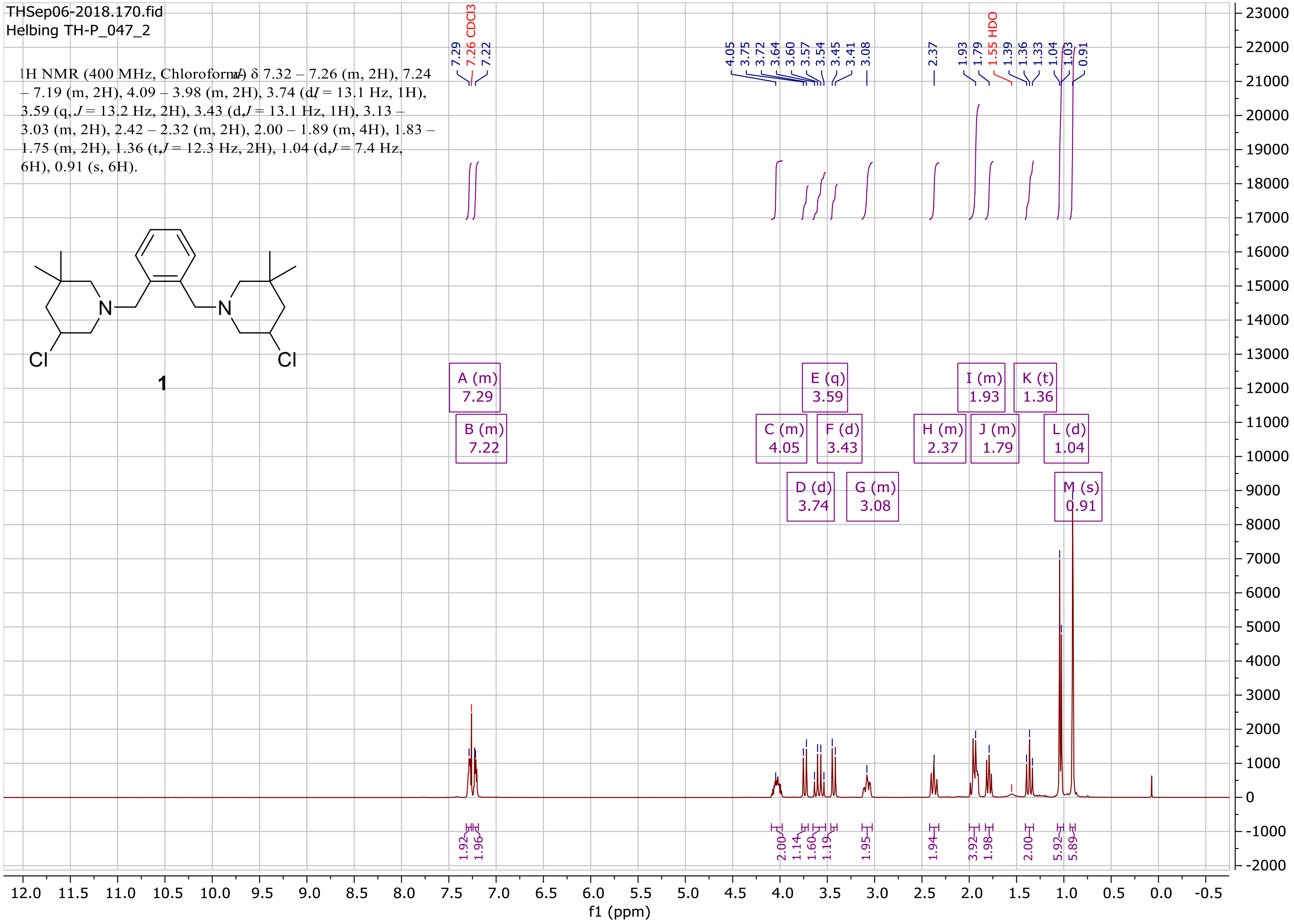
THSep06-2018.170.fid
Helbing TH-P_047_2
1H NMR (400 MHz, Chloroform-d) δ 7.32 – 7.26 (m, 2H), 7.24 – 7.19 (m, 2H), 4.09 – 3.98 (m, 2H), 3.74 (d, J = 13.1 Hz, 1H), 3.59 (q, J = 13.2 Hz, 2H), 3.43 (d, J = 13.1 Hz, 1H), 3.13 – 3.03 (m, 2H), 2.42 – 2.32 (m, 2H), 2.00 – 1.89 (m, 4H), 1.83 – 1.75 (m, 2H), 1.36 (t, J = 12.3 Hz, 2H), 1.04 (d, J = 7.4 Hz, 6H), 0.91 (s, 6H).
1
f1 (ppm)

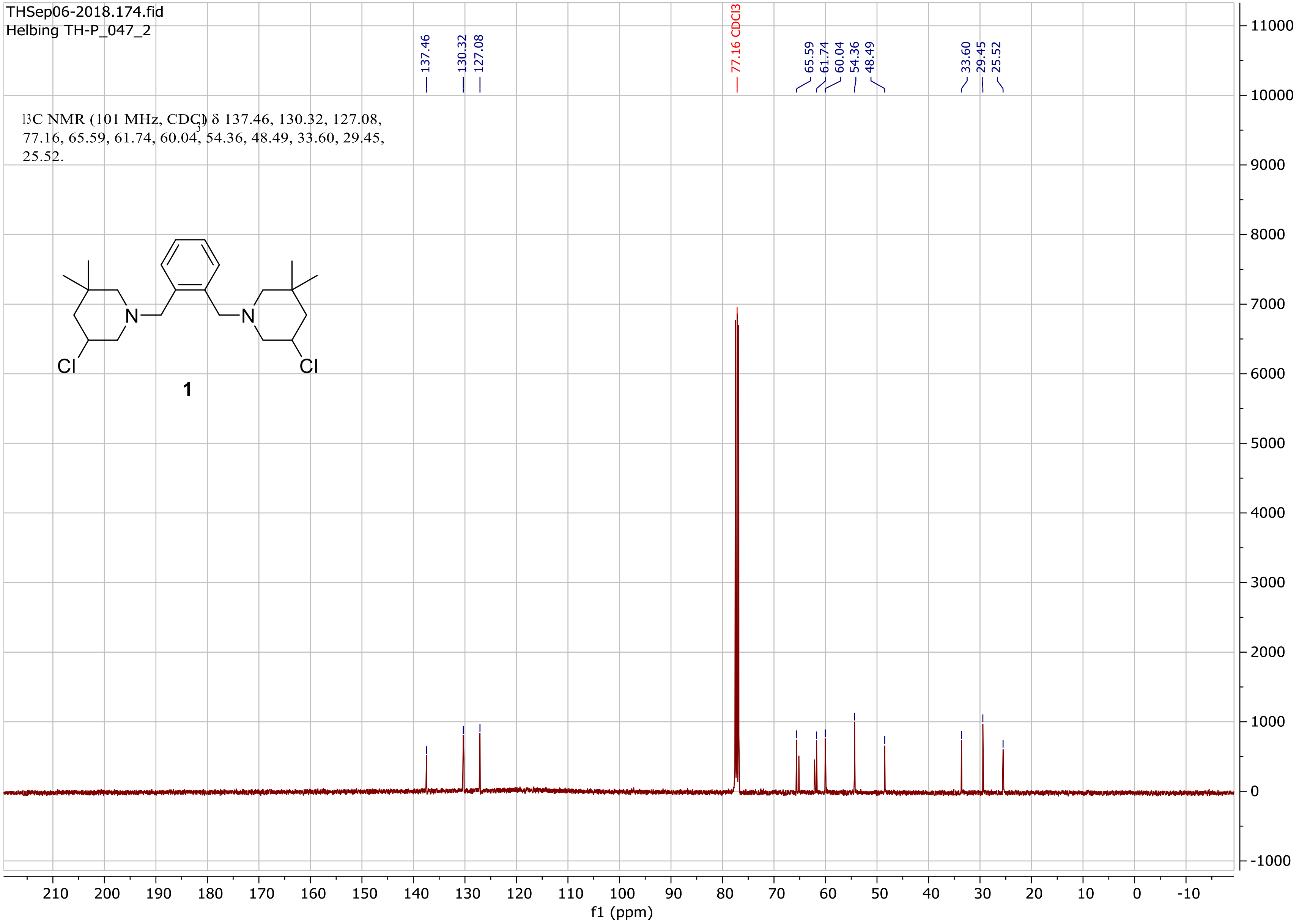
THSep06-2018.174.fid
Helbing TH-P_047_2
13C NMR (101 MHz, CDCl3) δ 137.46, 130.32, 127.08, 77.16, 65.59, 61.74, 60.04, 54.36, 48.49, 33.60, 29.45, 25.52.
137.46
130.32
127.08
77.16 CDCl3
65.59
61.74
60.04
54.36
48.49
33.60
29.45
25.52
N
N
Cl
Cl
1
f1 (ppm)

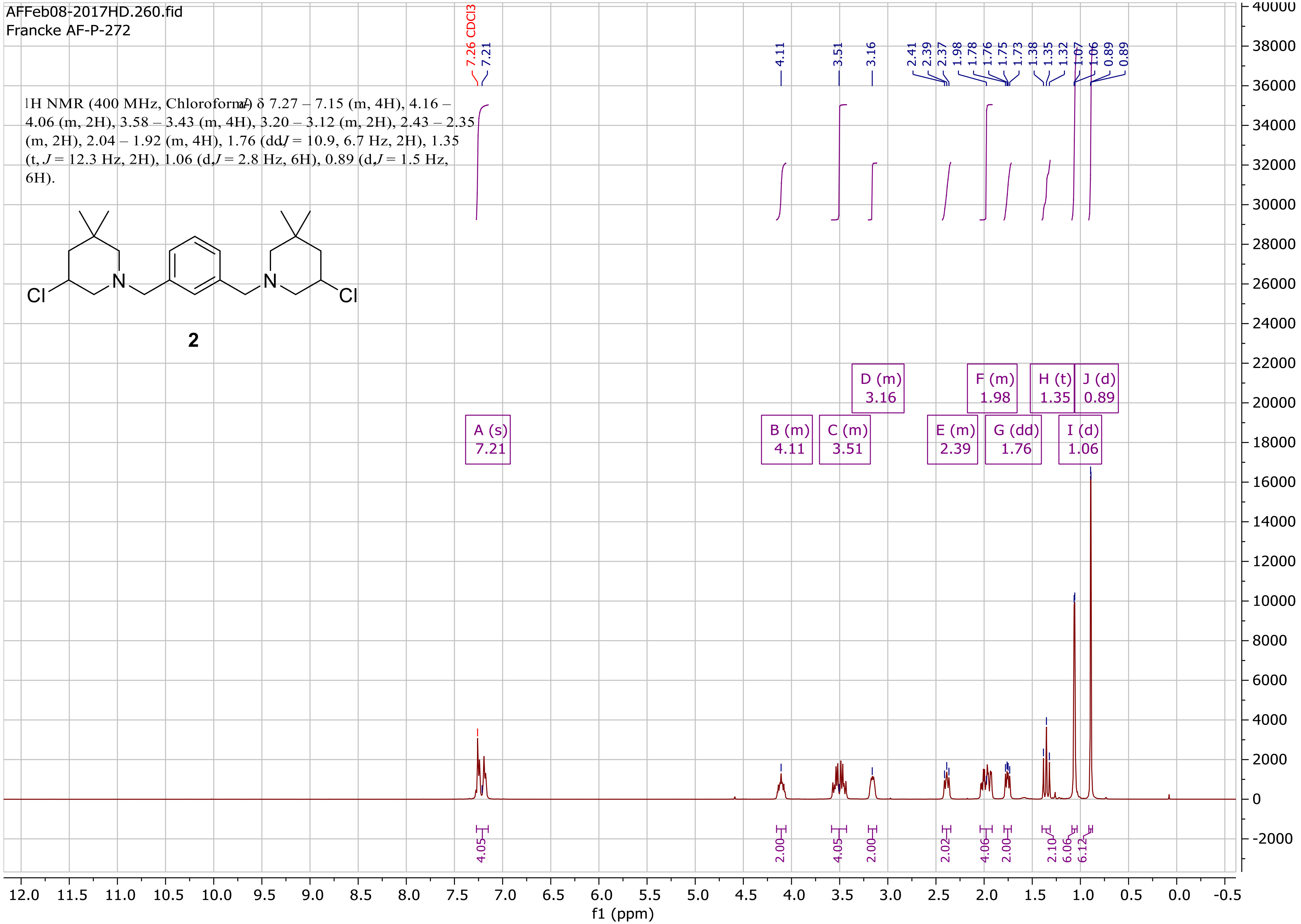
AFFeb08-2017HD.260.fid
Francke AF-P-272
1H NMR (400 MHz, Chloroform-d) δ 7.27 – 7.15 (m, 4H), 4.16 – 4.06 (m, 2H), 3.58 – 3.43 (m, 4H), 3.20 – 3.12 (m, 2H), 2.43 – 2.35 (m, 2H), 2.04 – 1.92 (m, 4H), 1.76 (dd, J = 10.9, 6.7 Hz, 2H), 1.35 (t, J = 12.3 Hz, 2H), 1.06 (d, J = 2.8 Hz, 6H), 0.89 (d, J = 1.5 Hz, 6H).
Cl
N
N
Cl
2
7.26 CDCl3
7.21
4.11
3.51
3.16
2.41
2.39
2.37
1.98
1.78
1.76
1.75
1.73
1.38
1.35
1.32
1.07
1.06
0.89
0.89
A (s) 7.21
B (m) 4.11
C (m) 3.51
D (m) 3.16
E (m) 2.39
F (m) 1.98
G (dd) 1.76
H (t) 1.35
I (d) 1.06
J (d) 0.89
4.05
2.00
4.05
2.00
2.02
4.06
2.00
2.10
6.06
6.12
f1 (ppm)

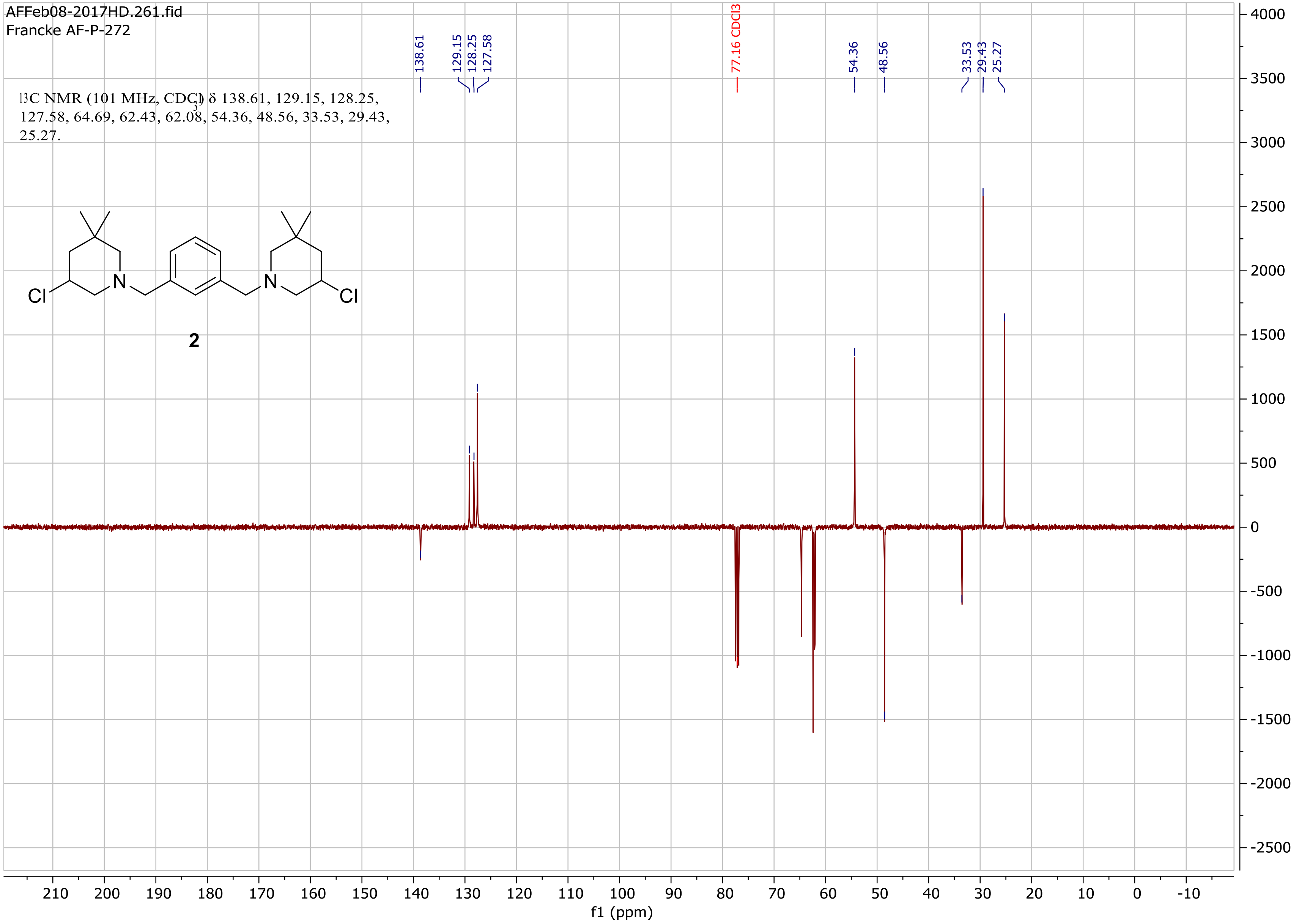
AFFeb08-2017HD.261.fid
Francke AF-P-272
13C NMR (101 MHz, CDCl3) δ 138.61, 129.15, 128.25, 127.58, 64.69, 62.43, 62.08, 54.36, 48.56, 33.53, 29.43, 25.27.
Cl
N
N
Cl
2
138.61
129.15
128.25
127.58
77.16 CDCl3
54.36
48.56
33.53
29.43
25.27
f1 (ppm)

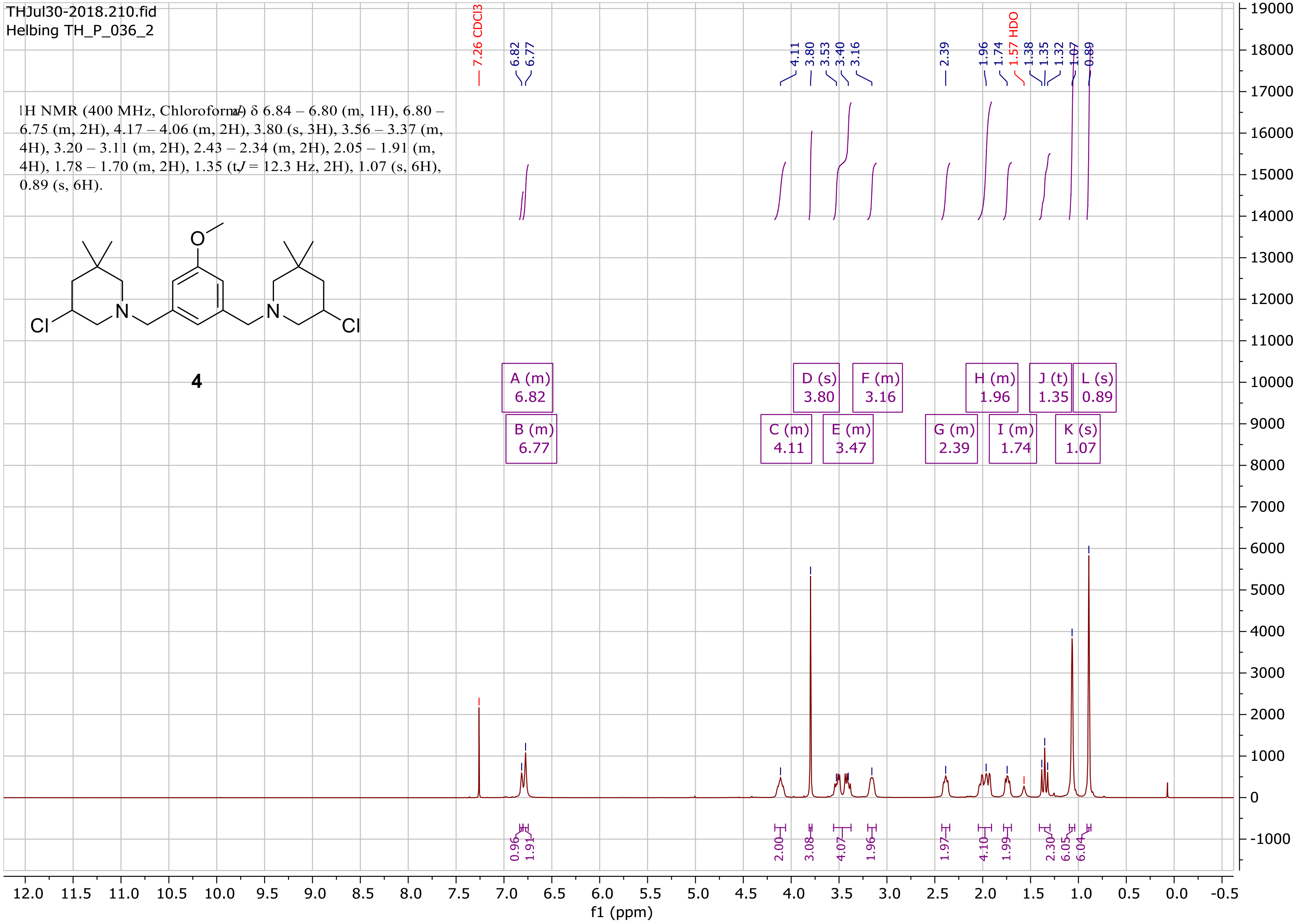
THJul30-2018.210.fid
Helbing TH_P_036_2
1H NMR (400 MHz, Chloroform-d) δ 6.84 – 6.80 (m, 1H), 6.80 – 6.75 (m, 2H), 4.17 – 4.06 (m, 2H), 3.80 (s, 3H), 3.56 – 3.37 (m, 4H), 3.20 – 3.11 (m, 2H), 2.43 – 2.34 (m, 2H), 2.05 – 1.91 (m, 4H), 1.78 – 1.70 (m, 2H), 1.35 (t, J = 12.3 Hz, 2H), 1.07 (s, 6H), 0.89 (s, 6H).
4
7.26 CDCl3
6.82
6.77
4.11
3.80
3.53
3.40
3.16
2.39
1.96
1.74
1.57 HDO
1.38
1.35
1.32
1.07
0.89
A (m) 6.82
B (m) 6.77
C (m) 4.11
D (s) 3.80
E (m) 3.47
F (m) 3.16
G (m) 2.39
H (m) 1.96
I (m) 1.74
J (t) 1.35
K (s) 1.07
L (s) 0.89
f1 (ppm)

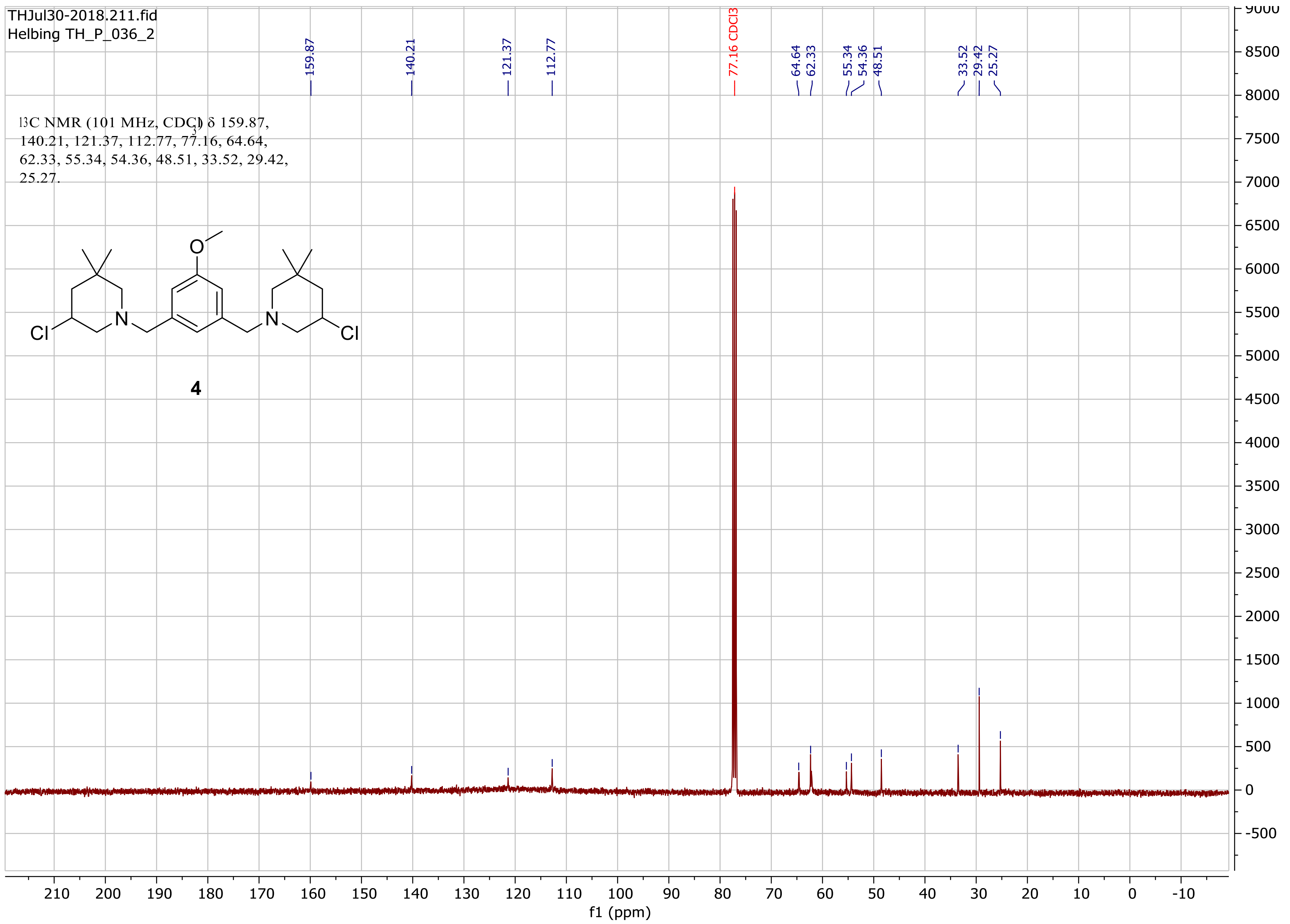
THJul30-2018.211.fid
Helbing TH_P_036_2
159.87
140.21
121.37
112.77
77.16 CDCl3
64.64
62.33
55.34
54.36
48.51
33.52
29.42
25.27
13C NMR (101 MHz, CDCl3) δ 159.87, 140.21, 121.37, 112.77, 77.16, 64.64, 62.33, 55.34, 54.36, 48.51, 33.52, 29.42, 25.27.
O
N
N
Cl
Cl
4
f1 (ppm)

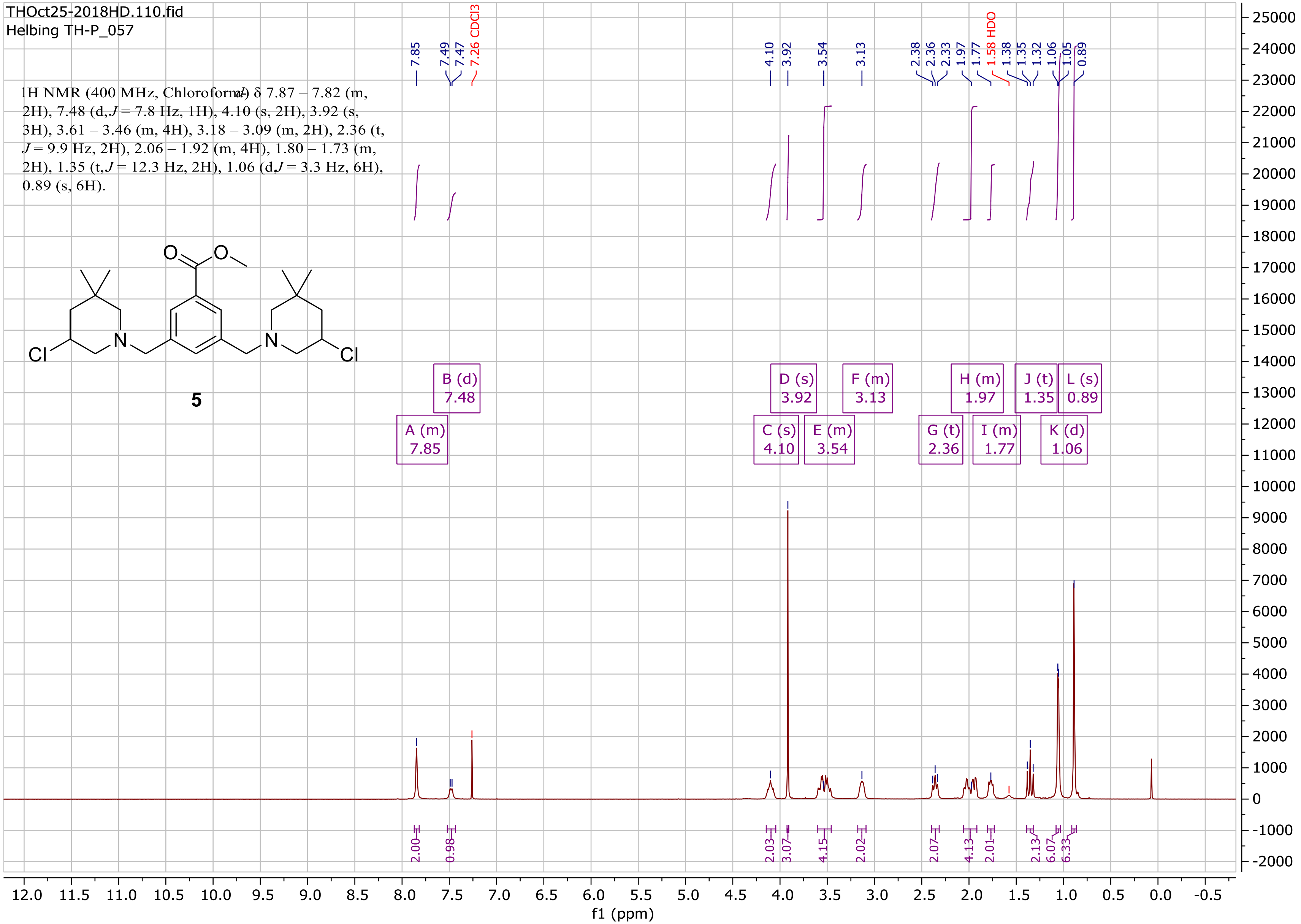
THOct25-2018HD.110.fid
Helbing TH-P_057
1H NMR (400 MHz, Chloroform-d) δ 7.87 – 7.82 (m, 2H), 7.48 (d, J = 7.8 Hz, 1H), 4.10 (s, 2H), 3.92 (s, 3H), 3.61 – 3.46 (m, 4H), 3.18 – 3.09 (m, 2H), 2.36 (t, J = 9.9 Hz, 2H), 2.06 – 1.92 (m, 4H), 1.80 – 1.73 (m, 2H), 1.35 (t, J = 12.3 Hz, 2H), 1.06 (d, J = 3.3 Hz, 6H), 0.89 (s, 6H).
5
A (m) 7.85
B (d) 7.48
C (s) 4.10
D (s) 3.92
E (m) 3.54
F (m) 3.13
G (t) 2.36
H (m) 1.97
I (m) 1.77
J (t) 1.35
K (d) 1.06
L (s) 0.89
7.26 CDCl3
1.58 HDO
f1 (ppm)

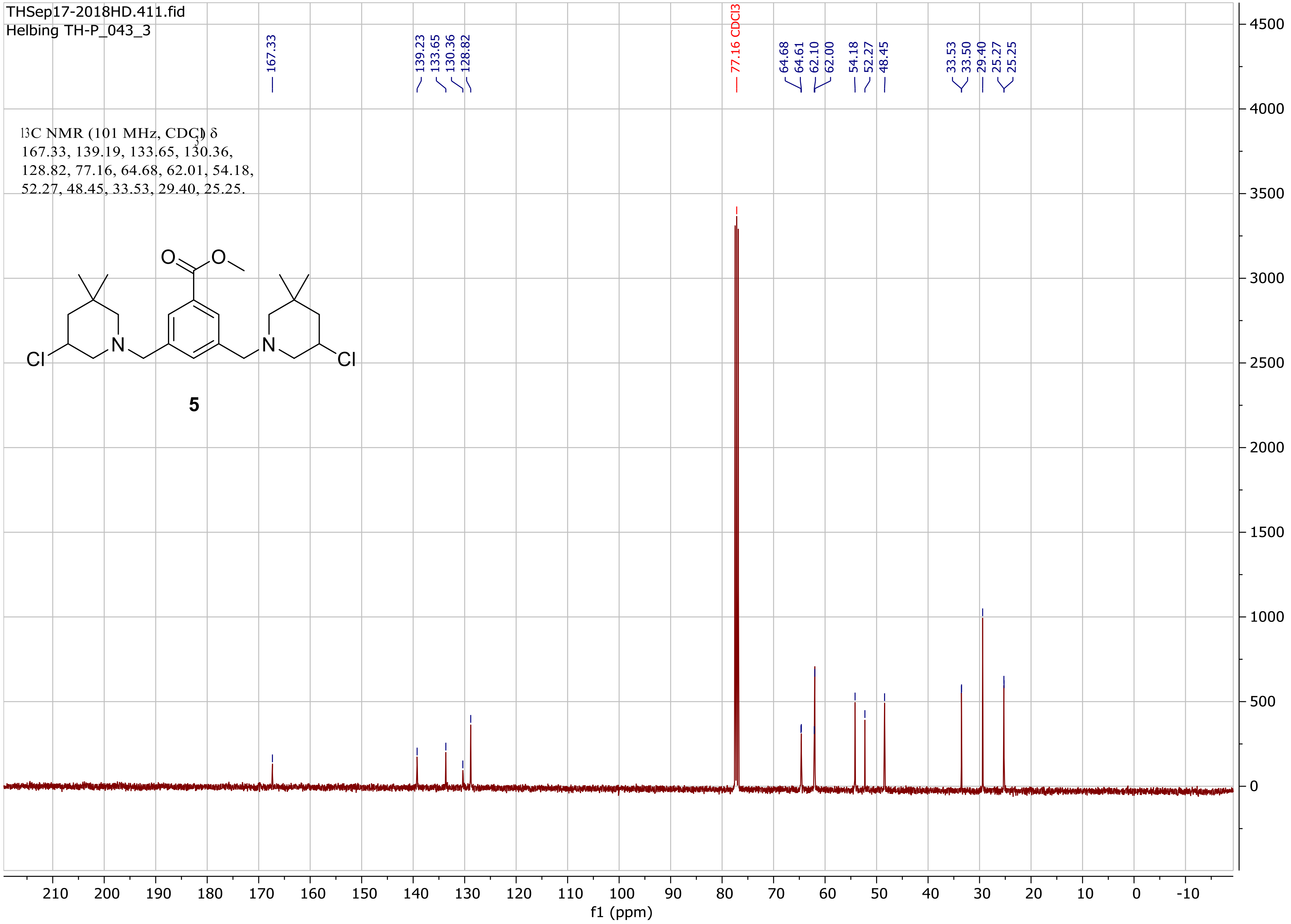
THSep17-2018HD.411.fid
Helbing TH-P_043_3
13C NMR (101 MHz, CDCl3) δ 167.33, 139.19, 133.65, 130.36, 128.82, 77.16, 64.68, 62.01, 54.18, 52.27, 48.45, 33.53, 29.40, 25.25.
167.33
139.23
133.65
130.36
128.82
77.16 CDCl3
64.68
64.61
62.10
62.00
54.18
52.27
48.45
33.53
33.50
29.40
25.27
25.25
5
f1 (ppm)

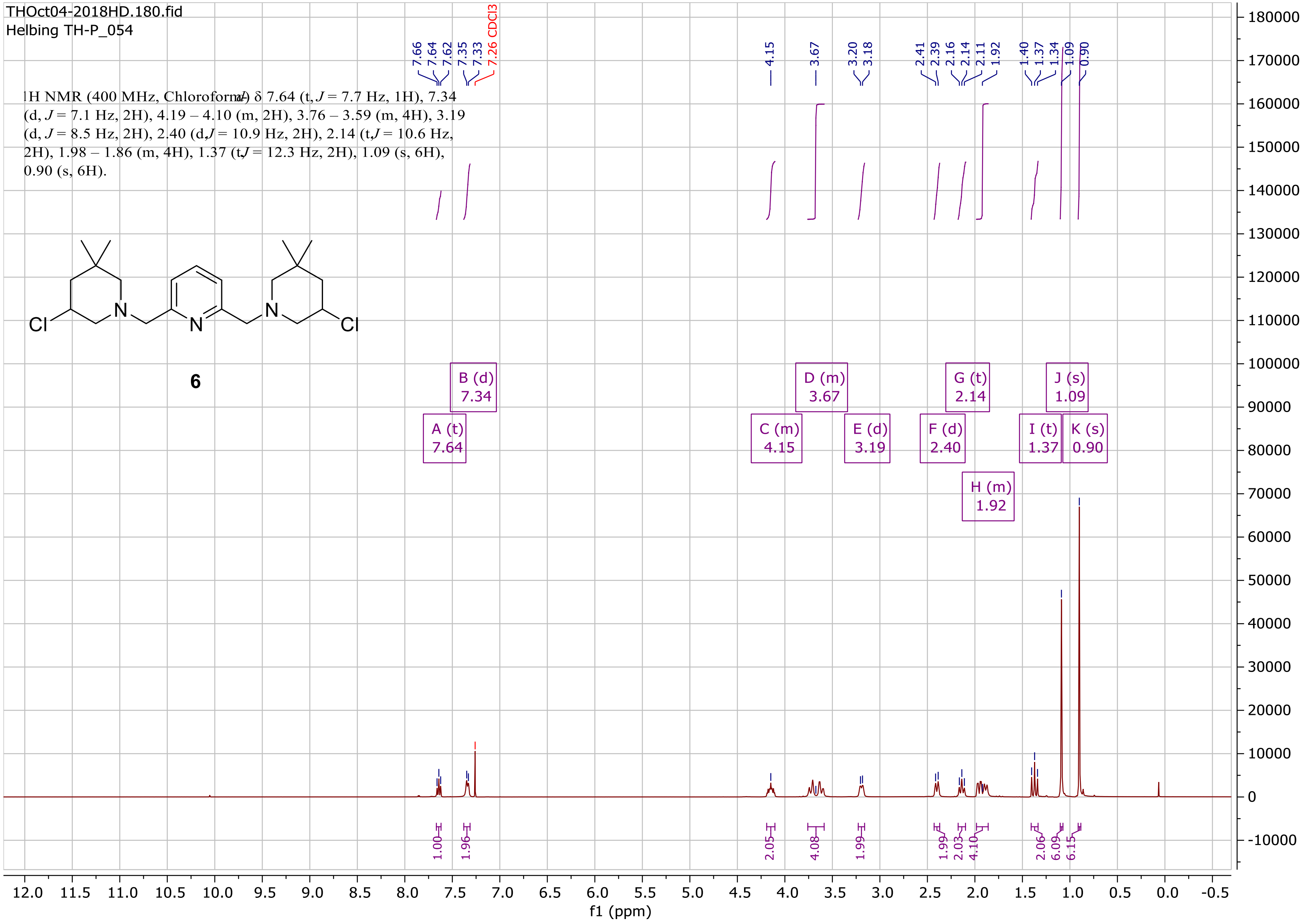
THOct04-2018HD.180.fid
Helbing TH-P_054
1H NMR (400 MHz, Chloroform-d) δ 7.64 (t, J = 7.7 Hz, 1H), 7.34 (d, J = 7.1 Hz, 2H), 4.19 – 4.10 (m, 2H), 3.76 – 3.59 (m, 4H), 3.19 (d, J = 8.5 Hz, 2H), 2.40 (d, J = 10.9 Hz, 2H), 2.14 (t, J = 10.6 Hz, 2H), 1.98 – 1.86 (m, 4H), 1.37 (t, J = 12.3 Hz, 2H), 1.09 (s, 6H), 0.90 (s, 6H).
Cl
N
N
N
Cl
6
7.66
7.64
7.62
7.35
7.33
7.26 CDCl3
4.15
3.67
3.20
3.18
2.41
2.39
2.16
2.14
2.11
1.92
1.40
1.37
1.34
1.09
0.90
A (t) 7.64
B (d) 7.34
C (m) 4.15
D (m) 3.67
E (d) 3.19
F (d) 2.40
G (t) 2.14
H (m) 1.92
I (t) 1.37
J (s) 1.09
K (s) 0.90
1.00
1.96
2.05
4.08
1.99
1.99
2.03
4.10
2.06
6.09
6.15
f1 (ppm)

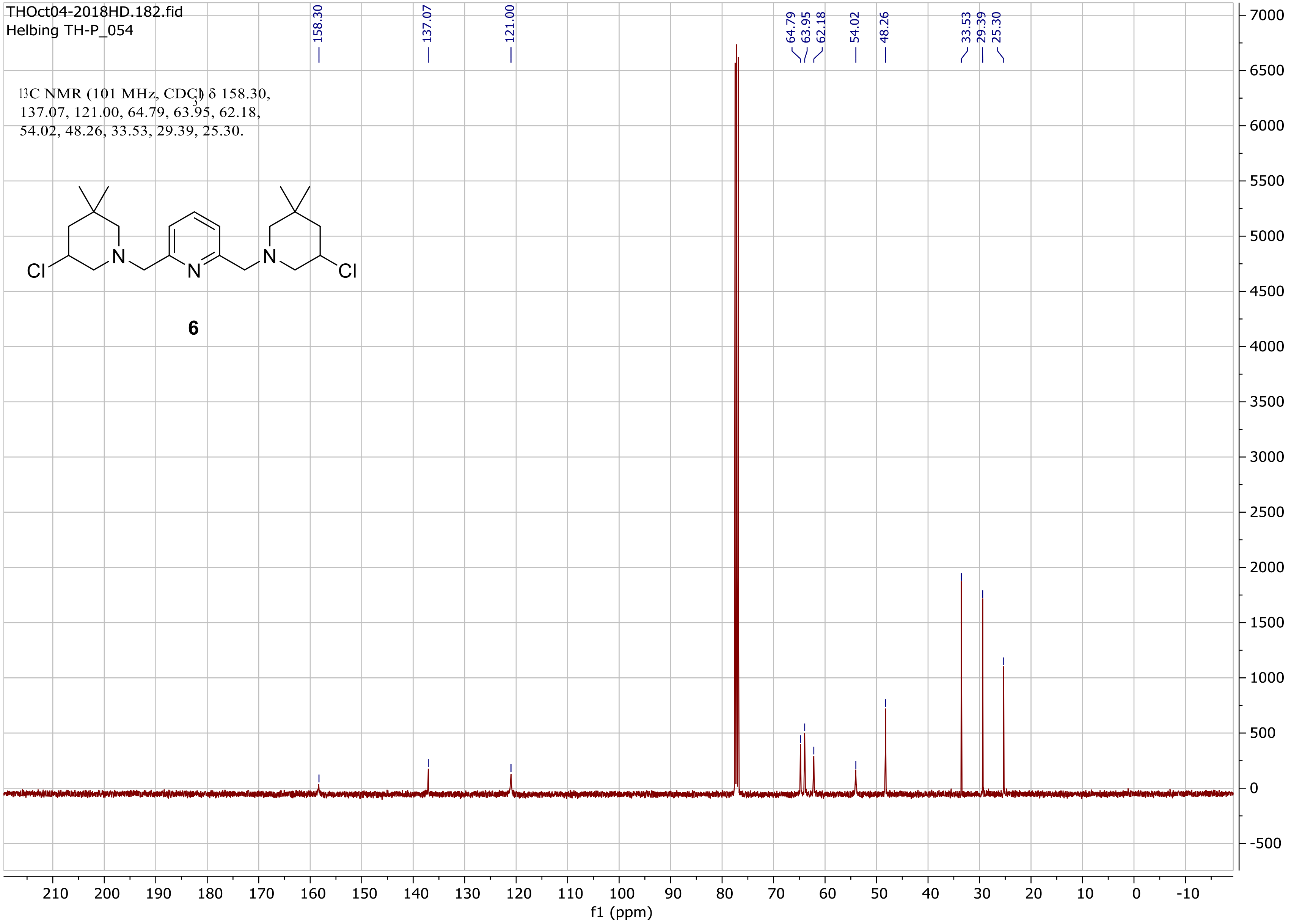
THOct04-2018HD.182.fid
Helbing TH-P_054
13C NMR (101 MHz, CDCl3) δ 158.30, 137.07, 121.00, 64.79, 63.95, 62.18, 54.02, 48.26, 33.53, 29.39, 25.30.
Cl
N
N
N
Cl
6
158.30
137.07
121.00
64.79
63.95
62.18
54.02
48.26
33.53
29.39
25.30
f1 (ppm)

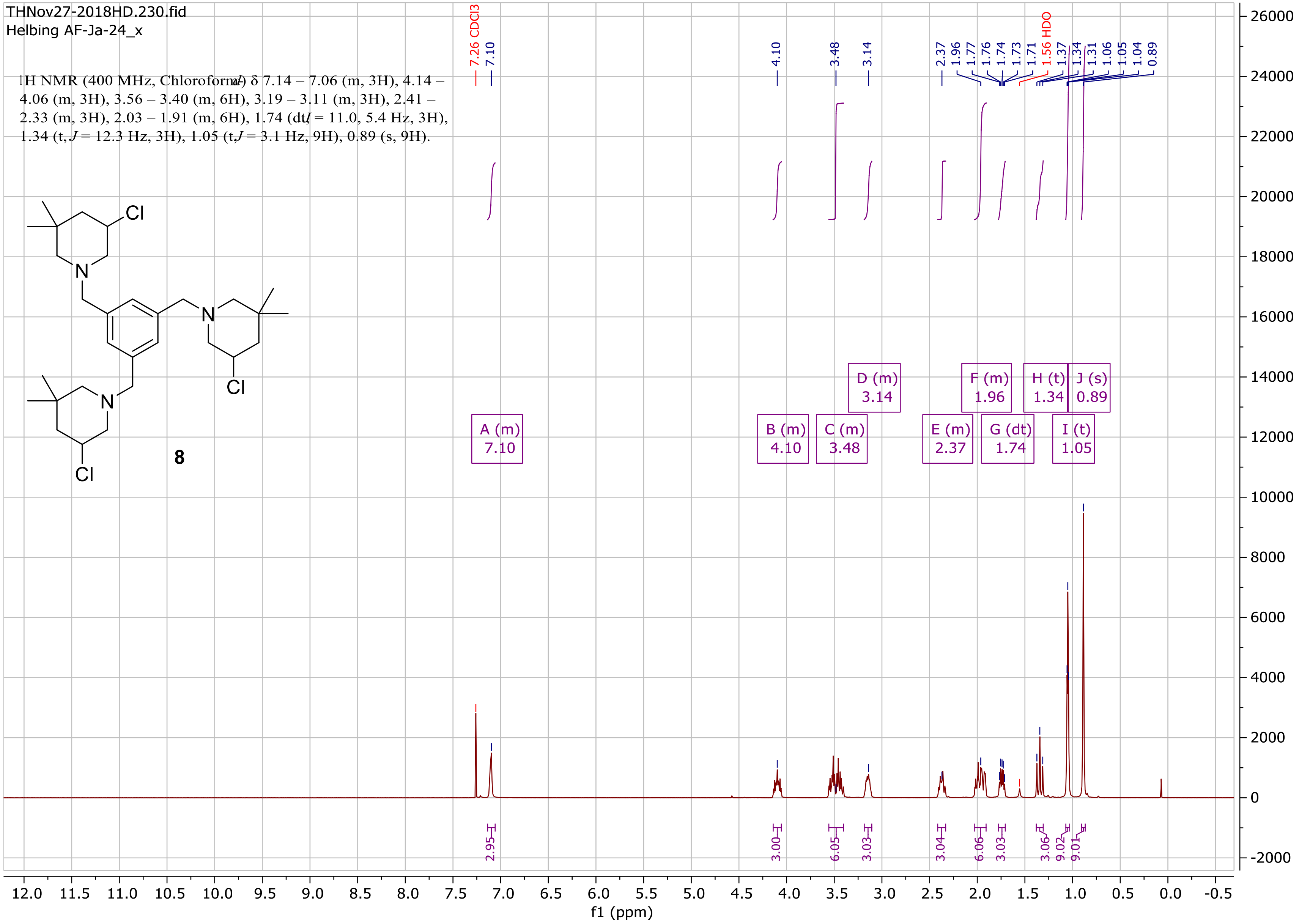
THNov27-2018HD.230.fid
Helbing AF-Ja-24_x
1H NMR (400 MHz, Chloroform-d) δ 7.14 – 7.06 (m, 3H), 4.14 – 4.06 (m, 3H), 3.56 – 3.40 (m, 6H), 3.19 – 3.11 (m, 3H), 2.41 – 2.33 (m, 3H), 2.03 – 1.91 (m, 6H), 1.74 (dt, J = 11.0, 5.4 Hz, 3H), 1.34 (t, J = 12.3 Hz, 3H), 1.05 (t, J = 3.1 Hz, 9H), 0.89 (s, 9H).
7.26 CDCl3
1.56 HDO
Cl
N
8
A (m) 7.10
B (m) 4.10
C (m) 3.48
D (m) 3.14
E (m) 2.37
F (m) 1.96
G (dt) 1.74
H (t) 1.34
I (t) 1.05
J (s) 0.89
f1 (ppm)

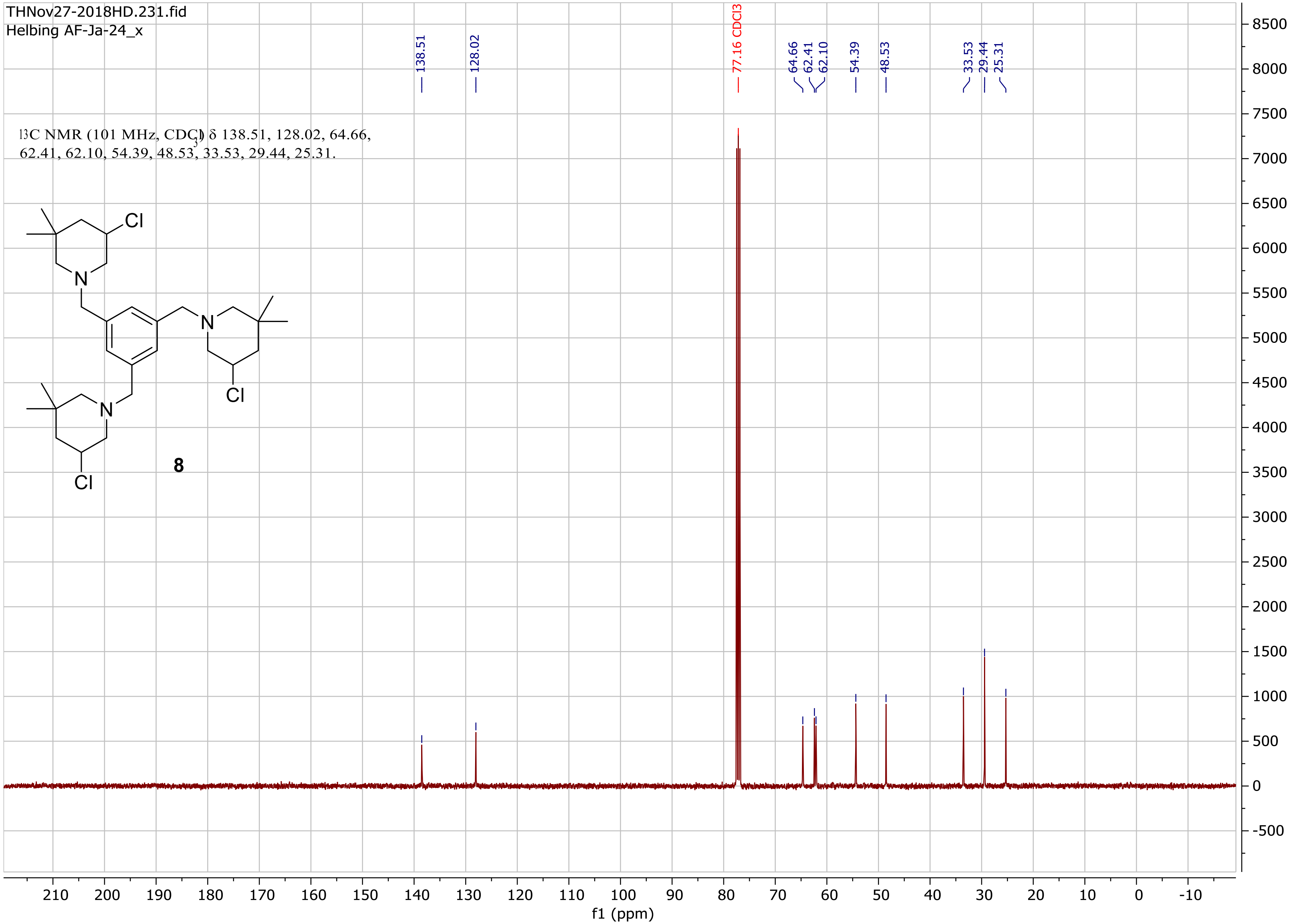
THNov27-2018HD.231.fid
Helbing AF-Ja-24_x
13C NMR (101 MHz, CDCl3) δ 138.51, 128.02, 64.66, 62.41, 62.10, 54.39, 48.53, 33.53, 29.44, 25.31.
138.51
128.02
77.16 CDCl3
64.66
62.41
62.10
54.39
48.53
33.53
29.44
25.31
Cl
N
8
f1 (ppm)
